# Binary and ternary charge-transfer complexes using 1,3,5-tri­nitro­benzene

**DOI:** 10.1107/S2056989018000245

**Published:** 2018-01-09

**Authors:** Tania Hill, Demetrius C. Levendis, Andreas Lemmerer

**Affiliations:** a Molecular Sciences Institute, School of Chemistry, University of the Witwatersrand, Private Bag, PO WITS, 2050, Johannesburg, South Africa

**Keywords:** crystal structure, charge transfer, ternary co-crystals

## Abstract

Three binary and one ternary charge-transfer complexes have been made using 1,3,5-tri­nitro­benzene, *viz*. 1,3,5-tri­nitro­benzene–2-acetylnaphthalene (1/1), C_6_H_3_N_3_O_6_·C_12_H_10_O, (I), 1,3,5-tri­nitro­benzene–9-bromo­anthracene (1/1), C_14_H_9_Br·C_6_H_3_N_3_O_6_, (II), 1,3,5-tri­nitro­benzene–methyl red (1/1), C_15_H_15_N_3_O_2_·C_6_H_3_N_3_O_6_, (III) (systematic name for methyl red: 2-{(*E*)-[4-(di­methyl­amino)­phen­yl]diazen­yl}benzoic acid), and 1,3,5-tri­nitro­benzene–1-naphthoic acid–2-amino-5-nitro­pyridine (1/1/1), C_6_H_3_N_3_O_6_·C_11_H_8_O_2_·C_5_H_5_N_3_O_2_, (IV). All charge-transfer complexes show alternating donor and acceptor stacks, which have weak C—H⋯O hydrogen bonds perpendicular to the stacking axis. In addition, complex (IV) is a crystal engineering attempt to modify the packing of the stacks by inserting a third mol­ecule into the structure. This third mol­ecule is stabilized by strong hydrogen bonds between the carb­oxy­lic acid group of the donor mol­ecule and the pyridine acceptor mol­ecule.

## Abstract 2

Three binary and one ternary charge-transfer complexes have been made using 1,3,5-tri­nitro­benzene, all of which show alternating donor and acceptor stacks, which have weak C—H⋯O hydrogen bonds perpendicular to the stacking axis. The final complex is a crystal engineering attempt to modify the packing of the stacks by inserting a third mol­ecule into the structure; this third mol­ecule features strong hydrogen bonds between the carb­oxy­lic acid group of the donor mol­ecule and the pyridine acceptor mol­ecule.

## Chemical context   

The crystal structure of 1,3,5-tri­nitro­benzene (TNB), an energetic or high-explosive material, was first reported as far back as 1930 (Hertel & Romer, 1930 ▸). A number of structures of pure TNB have appeared since then, including a neutron diffraction study in 1972 (Choi & Abel, 1972 ▸). More recently, polymorphs (Thallapally *et al.*, 2004 ▸) and pseudo-polymorphs of TNB (Jetti *et al.*, 2003 ▸) have been reported.

Crystal engineering, the conception and synthesis of mol­ecular solid-state structures, is fundamentally based upon the discernment and subsequent exploitation of inter­molecular inter­actions. Thus, primarily non-covalent bonding is used to achieve the organization of mol­ecules and ions in the solid state in order to produce materials with desired properties. The stability of an energetic material is one of the decisive factors in determining the viability of the final product, be it for fuels, propellants, pyrotechnics or explosives. If the energetic cannot be safely synthesized, handled and stored before its ultimate use, it is regarded as a failure, discarded and forgotten. Although not a forgotten but rather an old energetic material, 1,3,5-tri­nitro­benzene (TNB) falls into the class of energetics that are shock and heat sensitive, especially when in powdered dry form. Co-crystallization presents an opportunity to re-look at these problems for example, Guo *et al.* (2013*a* ▸) have shown that taking 2,4,6,8,10,12-hexa­nitro­hexa­aza­isowurtzitane (CL-20) and co-crystallizing it with caprolactam (CPL) has lead to new and inter­esting effects on the relevant properties. The importance of crystal engineering in the stabilization of explosive materials, such as tri­nitro­toluene (TNT) and ethyl­enedinitramine, was described recently (Landenberger *et al.*, 2010 ▸; Aakeröy *et al.*, 2015 ▸). Recently, Chen *et al.* (2017 ▸) isolated a novel co-crystal of 1,3,5-tri­nitro­benzene (TNB) and 1-nitro­naphthalene (NNAP), synthesized by using both solution and mechanochemical methods. The TNB/NNAP co-crystal has the largest proportion of π–π stacking inter­action (12.7%). A charge-transfer complex of TNT and TNB has also been reported (Guo *et al.*, 2013*b* ▸). The results indicate that the electronic effect has an influence on the inter­molecular inter­actions in the co-crystal. Our study comprises of an investigation of TNB as a model energetic with various polycyclic aromatic hydro­carbons in order to observe their effect on the structural aspects of the solid state. The structure and properties of many charge-transfer (CT) complexes of TNB with a variety of aromatic mol­ecules have been investigated (Brown *et al.*, 1964 ▸; Herbstein & Kaftory, 1975 ▸), and reviewed by Herbstein in different sections of his book (Herbstein, 2005 ▸). To this end, we have synthesized four new charge-transfer co-crystals, three binary (I)–(III), and one ternary (IV): tri­nitro­benzene–2-acetylnaphthalene, (I), tri­nitro­benzene–9-bromo­anthracene, (II), tri­nitro­benzene–methyl red, (III), and tri­nitro­benzene–1-naphthoic acid–2-amino-5-nitro­pyridine, (IV).
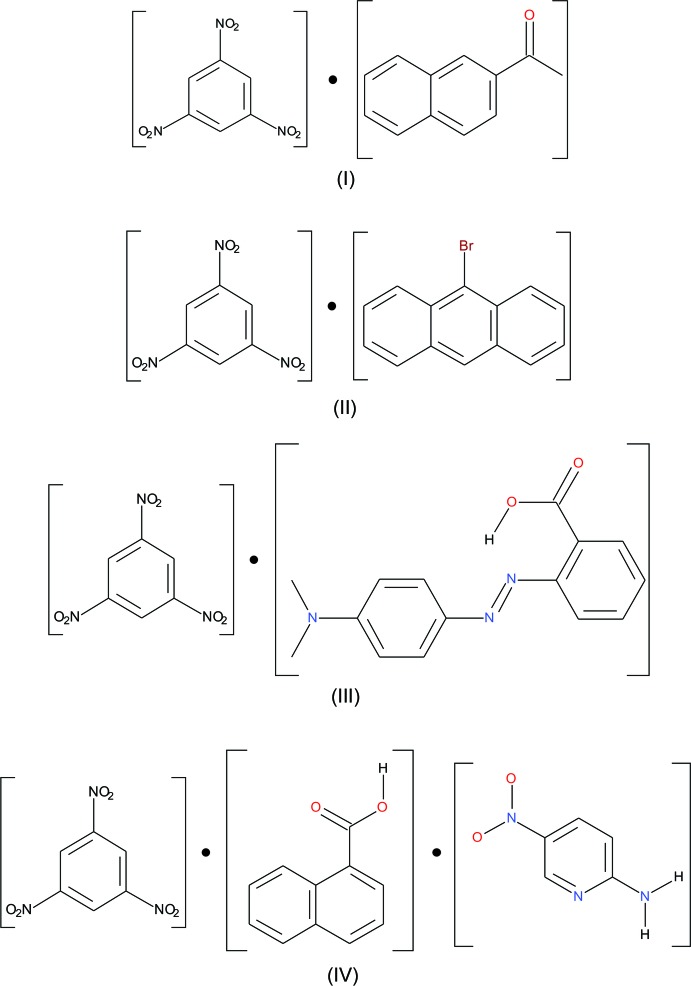


## Structural commentary   

**Figure 1:**
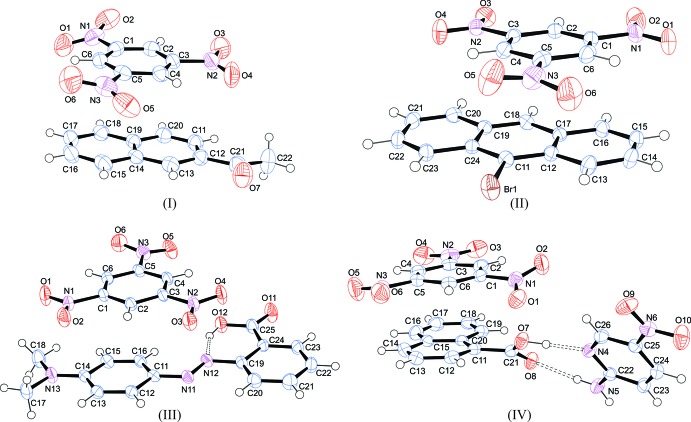
Perspective views of compounds (I)–(IV), showing the atom-numbering schemes. Displacement ellipsoids are drawn at the 50% probability level and H atoms are shown as small spheres of arbitrary radii. The dashed lines indicate the symmetry-independent N—H⋯O and O—H⋯N hydrogen bonds.

**Figure 2:**
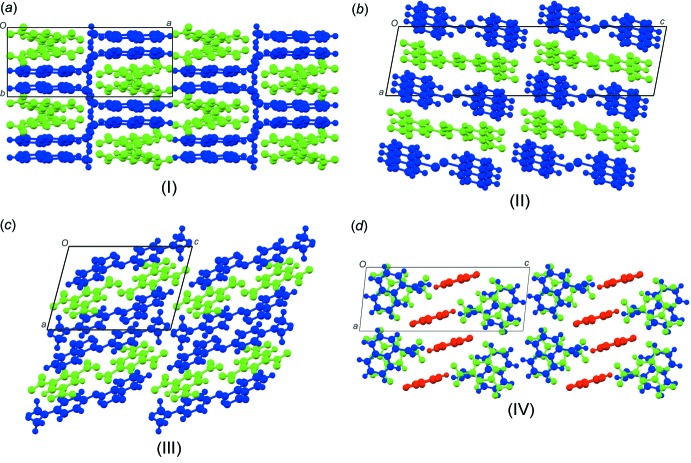
The packing diagrams for all four compounds (I)–(IV). The acceptor mol­ecules are shown in green and the donor mol­ecules in blue. The third mol­ecule of 2-amino-5-nitro­pyridine is shown in red in (IV).

**Figure 3:**
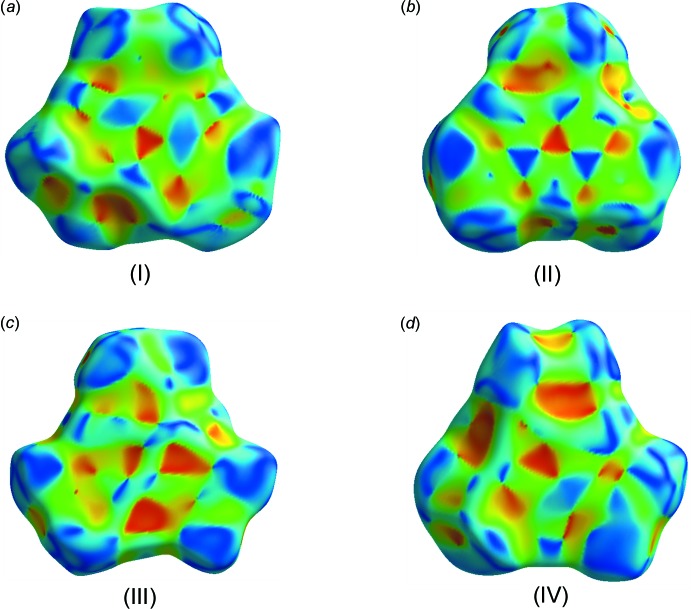
The mol­ecular Hirshfeld surfaces mapped with shape index for the TNB acceptor mol­ecule in (I)–(IV).

The asymmetric units and atom-labelling schemes for the title charge-transfer complexes are shown in Fig. 1 ▸. All compounds have *Z*′ = 1 with all mol­ecules in general positions. As a result of the strong polarizing effect of the nitro groups, TNB has an electron-poor π-system. On the other hand, the donor mol­ecules (polycyclic aromatic hydro­carbons) have an electron-rich π-system. The packing of the unit cell of the complexes follows a donor (*D*) acceptor (*A*) π–π inter­action, which is the major driving force in the formation of these complexes, as seen in Fig. 2 ▸ (donor mol­ecules shown in blue and acceptors in green), resulting in a general face-to-face π-stacking, with Table 1 ▸ summarizing the closest centroid–centroid distances between the TNB acceptor and aromatic donor systems. The inter­molecular inter­actions of the *D*⋯*A* stacks can be qu­anti­fied using Hirschfeld surface analysis as well as the resulting fingerprint plots using the programme *CrystalExplorer17.5* (Spackman & McKinnon, 2002 ▸). In the paper by Chen *et al.* (2017 ▸), the authors describe the regions of blue and red triangles on the Hirshfeld surface using the shape index as evidence of π–π inter­actions. Fig. 3 ▸ shows such surfaces plotted for the TNB mol­ecules in (I)–(IV). The red triangles show concave regions indicative of ring carbons of the π-stacked mol­ecule above it. (I) and (II) show the most triangles, indicative that they have the greatest proportion of π–π stacking of the four structures. This can be qu­anti­fied by looking at the contribution that C⋯C contacts make up in the fingerprint plots. (I) and (II) have values of 12.0 and 12.6%, respectively, much greater than the 4.4 and 7.5% for (III) and (IV), respectively. Table 2 ▸ summarizes the percentages of C⋯C, H⋯H and C⋯H contacts and the relevant fingerprint plots are given in the supporting information. In terms of the mol­ecular geometry, the TNB mol­ecules show some changes from the geometries encountered in the pure compound. The pure compound has torsion angles of the nitro group to the benzene ring in the range from 0 to 28.17°, whereas in the CT complexes described here they range from −20.0 (4) to +20.0 (5)°. The packing and hydrogen-bonding inter­actions are further described below individually for each compound.

## Supra­molecular features   

**Figure 4:**
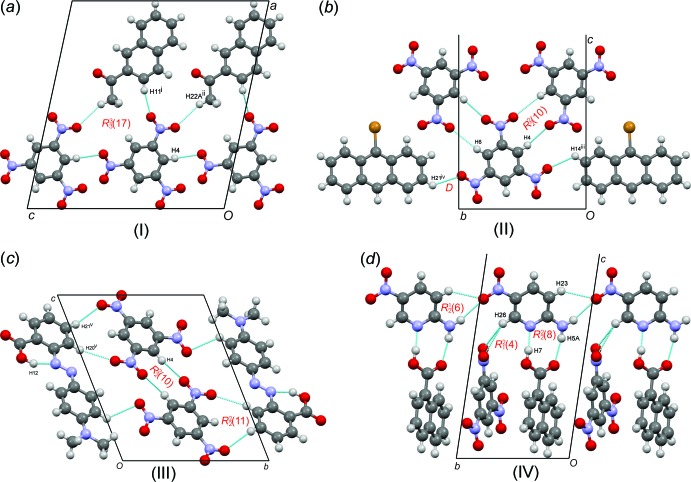
The hydrogen-bonding diagrams for all four compounds. Atoms with superscripts (i)–(v) are at the symmetry positions (−*x* + 1, −*y* + 1, −*z* + 1), (−*x* + 1, *y* − 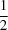, −*z* + 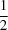), (*x*, *y* − 1, *z*), (*x* − 1, *y* + 1, *z*) and (1 − *x*, −*y*, −*z* + 1) respectively.

Structure (I) crystallizes in the *P*2_1_/*c* space group with both the TNB and 2-acetylnaphthalene mol­ecules in the asymmetric unit. The donor and acceptor mol­ecules pack in a checker-board fashion parallel to the *ab* plane (Fig. 2 ▸ *a*). In the plane perpendicular to the stacking, the *ac* plane, there are C—H⋯O inter­actions between TNB and 2-acetylnaphthalene mol­ecules (Table 3 ▸, Fig. 4 ▸ *a*), forming an 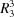
(17) ring described using graph set notation (Bernstein *et al.*, 1995 ▸).

Structure (II) crystallizes in the *P*2_1_/*c* space group with both the TNB and 9-bromo­anthracene in the asymmetric unit. The packing of the structure displays a clear separation of the donor (blue) and acceptor (green) layers (Fig. 2 ▸ *b*). The alternating *DA* stacks show that the bromine atom of the 9-bromo­anthracene packs in a head-to-head stacked fashion; however, the distance between the nearest Br atoms is very long [4.981 (1) Å], much longer than the sum of their van der Waals radii, and as a result Br⋯Br inter­actions are not involved in the mol­ecular aggregation. In the plane perpendicular to the stacking, there are C—H⋯O inter­actions (Table 4 ▸) between TNB mol­ecules forming an 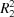
(10) ring, and a discrete hydrogen bond between the TNB and 9-bromo­anthracene mol­ecules (Fig. 4 ▸ *b*).

Structure (III) crystallizes in the *P* 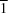 space group with both the TNB and the methyl red in the asymmetric unit. The packing of the structure illustrates that for each phenyl ring on the methyl red mol­ecule, there is an associated TNB mol­ecule. The two TNB mol­ecules are at different distances from the ring centroids, with a variation of *ca* 0.98 Å (Table 2 ▸). Along the *bc* plane, the TNB mol­ecules display similar hydrogen-bonded rings as those observed for (II) (Fig. 2 ▸ *c*), and an additional six-membered intra­molecular *S*(6) hydrogen bond is found (Fig. 1 ▸ *c*). The TNB and methyl red mol­ecules are again joined by C—H⋯O hydrogen bonds (Table 5 ▸) to the nitro oxygen atoms, forming an 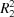
(11) ring (Fig. 4 ▸ *c*).

Structure (IV) crystallizes in the *P* 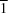 space group with the TNB, 1-naphthoic and 2-amino-5-nitro­pyridine mol­ecules in the asymmetric unit. The addition of a third mol­ecule into this charge-transfer complex results in groups of alternating *DA* stacks separated by the added pyridine component (Fig. 2 ▸ *d*). The TNB mol­ecules are joined by 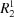
(6) rings, whereas a strong hydrogen-bonding inter­action between the *DA* stacks and the 2-amino-5-nitro­pyridine mol­ecule was found, forming an 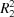
(8) ring, as well as a weaker bifurcated C—H⋯O 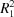
(4) ring (Table 6 ▸, Fig. 4 ▸ *d*); inter­estingly there is no additional *DA* stacking with the 1-naphthoic and the pyridine components.

In summary, we have contributed to the field of CT complexes using TNB, presenting further evidence that TNB is an ideal acceptor and, when paired with a donor that has hydrogen-bonding functionality, can be used to make ternary complexes.

## Database survey   

A database survey in the Cambridge Structural Database (CSD, Version 5.38; April 2017 update; Groom *et al.*, 2016 ▸) was undertaken for any structures containing the 1,3,5-tri­nitro­benzene moiety. A total of 135 hits where found, which was then reduced to 95 by evaluating if there is evidence of π–π inter­actions as indicative for a CT complex.

## Synthesis and crystallization   

All chemicals were purchased from commercial sources (Sigma Aldrich) and used as received without further purification. The 1,3,5-tri­nitro­benzene charge-transfer complexes were prepared in a 10 mL ethano­lic solution with a 1:1 or 1:1:1 stoichiometric ratio of the donor to the acceptor mol­ecule (based on 0.469 mmol of TNB), which was then heated until total dissolution took place (approx. 4 h). The solution was then cooled very slowly to obtain crystals suitable for X-ray diffraction. Detailed masses are as follows: (I): 0.100 g of 1,3,5-tri­nitro­benzene and 0.080 g of 2-acetyl­naphthalene; (II): 0.100 g of 1,3,5-tri­nitro­benzene and 0.121 g of 9-bromo­anthracene; (III): 0.100 g of 1,3,5-tri­nitro­benzene and 0.127 g of methyl red; and (IV): 0.100 g of 1,3,5-tri­nitro­benzene, 0.081 of 1-naphthoic acid and 0.065 g of 2-amino-5-nitro­pyridine.

## Refinement details   

Crystal data, data collection and structure refinement details are summarized in Table 7 ▸. For all compounds, the C-bound H atoms were placed geometrically [C—H bond lengths of 0.96 (methyl CH_3_), and 0.95 Å (Ar—H)] and refined as riding with *U*_iso_(H) = 1.2*U*_eq_(Ar-C) or *U*_iso_(H) = 1.5*U*_eq_(methyl-C). The O and N–bound H atoms were located in the difference map and their coordinates and isotropic displacement parameters allowed to refine freely.

## Supplementary Material

Crystal structure: contains datablock(s) I, II, III, IV, shelx. DOI: 10.1107/S2056989018000245/eb2004sup1.cif

Structure factors: contains datablock(s) I. DOI: 10.1107/S2056989018000245/eb2004Isup2.hkl

Structure factors: contains datablock(s) II. DOI: 10.1107/S2056989018000245/eb2004IIsup3.hkl

Structure factors: contains datablock(s) III. DOI: 10.1107/S2056989018000245/eb2004IIIsup4.hkl

Structure factors: contains datablock(s) IV. DOI: 10.1107/S2056989018000245/eb2004IVsup5.hkl

Fingerprint plots. DOI: 10.1107/S2056989018000245/eb2004sup6.pdf

Supporting information file. DOI: 10.1107/S2056989018000245/eb2004Isup7.cml

Supporting information file. DOI: 10.1107/S2056989018000245/eb2004IIsup8.cml

Supporting information file. DOI: 10.1107/S2056989018000245/eb2004IIIsup9.cml

Supporting information file. DOI: 10.1107/S2056989018000245/eb2004IVsup10.cml

CCDC references: 1814637, 1814636, 1814635, 1814634

Additional supporting information:  crystallographic information; 3D view; checkCIF report

## Figures and Tables

**Table 1:** Centroid distances (Å) between the tri­nitro­benzene and the ring centroids (*Cg*) of the aromatic polycyclics

Structure	Donor	Acceptor	*Cg*⋯*Cg*	Symmetry operator
(I)	C1–C6	C11–C20	3.3745 (2)	*x*, *y*, *z*
(II)	C1–C6	C11–C24	3.5173 (11)	*x*, *y*, *z*
(III)	C1–C6	C11–C16	3.6587 (14)	1 − *x*, 1 − *y*, 1 − *z*
(III)	C1–C6	C19–C24	4.6432 (18)	*x*, *y*, *z*
(IV)	C1–C6	C11–C20	4.0417 (8)	*x*, *y* + 1, *z*

**Table 2:** Proportion (%) of inter­molecular contacts between donor and acceptor mol­ecules in the Hirshfeld fingerprint plots

Structure	C⋯C	H⋯H	C⋯H
(I)	12.0	10.7	1.5
(II)	12.6	6.6	0.9
(III)	4.4	11.0	5.4
(IV)	7.5	8.8	4.6

**Table 3:** Hydrogen-bond geometry (Å, °) for (I)

*D*—H⋯*A*	*D*—H	H⋯*A*	*D*⋯*A*	*D*—H⋯*A*
C4—H4⋯O2^i^	0.93	2.4	3.189 (3)	143
C11—H11⋯O3^ii^	0.93	2.48	3.323 (4)	150
C22—H22*A*⋯O4^iii^	0.96	2.64	3.554 (4)	159

Symmetry codes: (i) 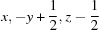; (ii) 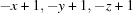; (iii) 
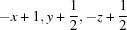
.

**Table 4:** Hydrogen-bond geometry (Å, °) for (II)

*D*—H⋯*A*	*D*—H	H⋯*A*	*D*⋯*A*	*D*—H⋯*A*
C4—H4⋯O6^i^	0.95	2.5	3.287 (3)	140
C6—H6⋯O5^ii^	0.95	2.68	3.593 (3)	162
C14—H14⋯O4^iii^	0.95	2.57	3.255 (3)	129
C21—H21⋯O1^iv^	0.95	2.65	3.364 (3)	132

Symmetry codes: (i) 
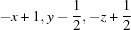
; (ii) 
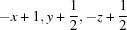
; (iii) 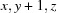; (iv) 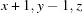.

**Table 5:** Hydrogen-bond geometry (Å, °) for (III)

*D*—H⋯*A*	*D*—H	H⋯*A*	*D*⋯*A*	*D*—H⋯*A*
C4—H4⋯O4^i^	0.95	2.35	3.285 (4)	169
C15—H15⋯O11^ii^	0.95	2.34	3.254 (4)	161
C18—H18*A*⋯O11^ii^	0.98	2.55	3.519 (4)	170
C20—H20⋯O3^iii^	0.95	2.64	3.574 (4)	167
O12—H12⋯N12	0.95 (6)	1.70 (6)	2.577 (3)	153 (5)
C21—H21⋯O2^iii^	0.95	2.56	3.469 (4)	161

Symmetry codes: (i) 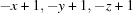; (ii) 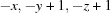; (iii) 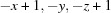.

**Table 6:** Hydrogen-bond geometry (Å, °) for (IV)

*D*—H⋯*A*	*D*—H	H⋯*A*	*D*⋯*A*	*D*—H⋯*A*
N5—H5*A*⋯O8	0.90 (5)	2.03 (5)	2.919 (5)	168 (5)
N5—H5*B*⋯O9^i^	0.86 (4)	2.24 (4)	3.069 (5)	161 (4)
O7—H7⋯N4	0.95 (5)	1.71 (5)	2.650 (4)	171 (5)
C23—H23⋯O9^i^	0.95	2.5	3.272 (5)	139
C26—H26⋯O1	0.95	2.69	3.530 (5)	147
C26—H26⋯O2	0.95	2.72	3.477 (5)	138

Symmetry code: (i) 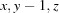.

**Table 7:** Experimental details

	(I)	(II)	(III)	(IV)
Crystal data				
Chemical formula	C_6_H_3_N_3_O_6_·C_12_H_10_O	C_14_H_9_Br·C_6_H_3_N_3_O_6_	C_15_H_15_N_3_O_2_·C_6_H_3_N_3_O_6_	C_6_H_3_N_3_O_6_·C_11_H_8_O_2_·C_5_H_5_N_3_O_2_
*M*_r_	383.31	470.24	482.41	524.41
Crystal system, space group	Monoclinic, *P*2_1_/*c*	Monoclinic, *P*2_1_/*c*	Triclinic, *P* 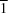	Triclinic, *P* 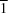
Temperature (K)	293	173	173	173
*a*, *b*, *c* (Å)	16.6728 (10), 6.8197 (3), 15.4419 (7)	7.0928 (2), 9.7701 (3), 27.0563 (7)	8.550 (3), 10.437 (3), 13.072 (5)	7.5365 (15), 7.9003 (16), 19.153 (4)
α, β, γ (°)	90, 102.217 (3), 90	90, 100.674 (1), 90	110.689 (10), 103.510 (12), 90.730 (12)	97.580 (7), 94.667 (6), 99.547 (7)
*V* (Å^3^)	1716.03 (15)	1842.49 (9)	1055.2 (7)	1108.5 (4)
*Z*	4	4	2	2
Radiation type	Mo *K*α	Mo *K*α	Mo *K*α	Mo *K*α
μ (mm^−1^)	0.12	2.28	0.12	0.13
Crystal size (mm)	0.15 × 0.12 × 0.07	0.33 × 0.12 × 0.11	0.29 × 0.13 × 0.12	0.63 × 0.33 × 0.06

Data collection				
Diffractometer	Bruker D8 Venture Photon CCD area detector	Bruker D8 Venture Photon CCD area detector	Bruker D8 Venture Photon CCD area detector	Bruker D8 Venture Photon CCD area detector
Absorption correction	Multi-scan (*SADABS*; Sheldrick, 1996 ▸)	Multi-scan (*SADABS*; Sheldrick, 1996 ▸)	Multi-scan (*SADABS*; Sheldrick, 1996 ▸)	Multi-scan (*SADABS*; Sheldrick, 1996 ▸)
*T*_min_, *T*_max_	0.9, 0.95	0.56, 0.77	0.9, 0.95	0.9, 0.95
No. of measured, independent and observed [*I* > 2σ(*I*)] reflections	15612, 3195, 1874	28424, 4450, 3758	39527, 5072, 3262	12982, 3988, 3368
*R*_int_	0.063	0.048	0.072	0.034

Refinement				
*R*[*F* ^2^ > 2σ(*F* ^2^)], *wR*(*F* ^2^), *S*	0.053, 0.152, 1.01	0.040, 0.096, 1.08	0.078, 0.249, 1.07	0.072, 0.168, 1.22
No. of reflections	3195	4450	5072	3988
No. of parameters	254	271	322	355
H-atom treatment	H-atom parameters constrained	H-atom parameters constrained	H atoms treated by a mixture of independent and constrained refinement	H atoms treated by a mixture of independent and constrained refinement
Δρ_max_, Δρ_min_ (e Å^−3^)	0.40, −0.29	1.42, −0.94	0.49, −0.43	0.32, −0.37

Computer programs: *APEX3*, *SAINT-Plus* and *XPREP* (Bruker, 2016 ▸), *SHELXS97* (Sheldrick, 2015 ▸), *SHELXL2017/1* (Sheldrick, 2015 ▸), *ORTEP-3 for Windows* and *WinGX* publication routines (Farrugia, 2012 ▸) and *Mercury* (Macrae *et al.*, 2006 ▸).

## supplementary crystallographic information

### 1,3,5-Trinitrobenzene–1-(naphthalen-2-yl)ethan-1-one (1/1) (I) Crystal data

**Table:**

C_6_H_3_N_3_O_6_·C_12_H_10_O	*F*(000) = 792
*M**_r_* = 383.31	*D*_x_ = 1.484 Mg m^−^^3^
Monoclinic, *P*2_1_/*c*	Mo *K*α radiation, λ = 0.71073 Å
Hall symbol: -P 2ybc	Cell parameters from 1787 reflections
*a* = 16.6728 (10) Å	θ = 2.5–19.9°
*b* = 6.8197 (3) Å	µ = 0.12 mm^−^^1^
*c* = 15.4419 (7) Å	*T* = 293 K
β = 102.217 (3)°	Plate, yellow
*V* = 1716.03 (15) Å^3^	0.15 × 0.12 × 0.07 mm
*Z* = 4	

### 1,3,5-Trinitrobenzene–1-(naphthalen-2-yl)ethan-1-one (1/1) (I) Data collection

**Table:**

Bruker D8 Venture Photon CCD area detector diffractometer	1874 reflections with *I* > 2σ(*I*)
Graphite monochromator	*R*_int_ = 0.063
ω scans	θ_max_ = 25.5°, θ_min_ = 2.5°
Absorption correction: multi-scan (SADABS; Sheldrick, 1996)	*h* = −18→20
*T*_min_ = 0.9, *T*_max_ = 0.95	*k* = −8→8
15612 measured reflections	*l* = −18→18
3195 independent reflections	

### 1,3,5-Trinitrobenzene–1-(naphthalen-2-yl)ethan-1-one (1/1) (I) Refinement

**Table:**

Refinement on *F*^2^	0 constraints
Least-squares matrix: full	Hydrogen site location: inferred from neighbouring sites
*R*[*F*^2^ > 2σ(*F*^2^)] = 0.053	H-atom parameters constrained
*wR*(*F*^2^) = 0.152	*w* = 1/[σ^2^(*F*_o_^2^) + (0.0652*P*)^2^ + 0.4622*P*] where *P* = (*F*_o_^2^ + 2*F*_c_^2^)/3
*S* = 1.01	(Δ/σ)_max_ < 0.001
3195 reflections	Δρ_max_ = 0.40 e Å^−^^3^
254 parameters	Δρ_min_ = −0.28 e Å^−^^3^
0 restraints	

### 1,3,5-Trinitrobenzene–1-(naphthalen-2-yl)ethan-1-one (1/1) (I) Special details

**Table:**

Experimental. (SADABS; Sheldrick, 1996)
Geometry. All standard uncertainties (s.u.) (except the s.u. in the dihedral angle between two l.s. planes) are estimated using the full covariance matrix. The cell s.u. are taken into account individually in the estimation of s.u. in distances, angles and torsion angles; correlations between s.u. in cell parameters are only used when they are defined by crystal symmetry. An approximate (isotropic) treatment of cell s.u. is used for estimating s.u. involving l.s. planes.

### 1,3,5-Trinitrobenzene–1-(naphthalen-2-yl)ethan-1-one (1/1) (I) Fractional atomic coordinates and isotropic or equivalent isotropic displacement parameters (Å^2^)

**Table:**

	*x*	*y*	*z*	*U*_iso_*/*U*_eq_	
C1	0.21511 (18)	0.3392 (4)	0.53179 (17)	0.0389 (7)	
C2	0.29340 (17)	0.3382 (4)	0.51644 (18)	0.0405 (7)	
H2	0.338939	0.313665	0.561495	0.049*	
C3	0.30078 (16)	0.3753 (4)	0.43143 (18)	0.0375 (6)	
C4	0.23515 (17)	0.4104 (3)	0.36307 (17)	0.0380 (7)	
H4	0.242162	0.435636	0.305949	0.046*	
C5	0.15851 (17)	0.4066 (3)	0.38276 (17)	0.0373 (6)	
C6	0.14629 (17)	0.3724 (3)	0.46663 (17)	0.0393 (7)	
H6	0.093996	0.371735	0.478805	0.047*	
N1	0.2053 (2)	0.3015 (4)	0.62306 (17)	0.0552 (7)	
N2	0.38409 (17)	0.3748 (3)	0.4122 (2)	0.0530 (7)	
N3	0.08572 (17)	0.4407 (4)	0.31102 (18)	0.0552 (7)	
O1	0.14078 (18)	0.3516 (4)	0.64216 (16)	0.0780 (8)	
O2	0.26172 (18)	0.2195 (3)	0.67273 (14)	0.0753 (8)	
O3	0.44113 (14)	0.3279 (3)	0.47247 (19)	0.0778 (8)	
O4	0.39003 (15)	0.4197 (4)	0.33769 (17)	0.0748 (7)	
O5	0.09752 (15)	0.5061 (3)	0.24181 (15)	0.0707 (7)	
O6	0.01788 (16)	0.4066 (4)	0.32863 (18)	0.0871 (8)	
C11	0.37425 (18)	0.8616 (4)	0.44847 (17)	0.0416 (7)	
H11	0.431199	0.855588	0.46446	0.05*	
C12	0.33560 (17)	0.8871 (4)	0.35789 (17)	0.0386 (6)	
C13	0.25164 (17)	0.9004 (4)	0.33542 (18)	0.0399 (7)	
H13	0.225873	0.918682	0.276412	0.048*	
C14	0.20357 (16)	0.8866 (3)	0.40060 (17)	0.0355 (6)	
C15	0.11648 (17)	0.8960 (4)	0.37776 (19)	0.0447 (7)	
H15	0.090008	0.917791	0.319232	0.054*	
C16	0.07171 (19)	0.8732 (4)	0.4412 (2)	0.0491 (8)	
H16	0.014733	0.878211	0.425528	0.059*	
C17	0.11022 (19)	0.8425 (4)	0.5296 (2)	0.0490 (8)	
H17	0.078694	0.827225	0.572177	0.059*	
C18	0.19420 (19)	0.8348 (4)	0.55427 (19)	0.0437 (7)	
H18	0.2192	0.815473	0.61345	0.052*	
C19	0.24309 (17)	0.8560 (3)	0.49009 (17)	0.0363 (6)	
C20	0.32950 (17)	0.8457 (4)	0.51201 (18)	0.0402 (7)	
H20	0.356146	0.827898	0.57077	0.048*	
C21	0.3838 (2)	0.8968 (4)	0.2863 (2)	0.0473 (7)	
C22	0.4755 (2)	0.9057 (5)	0.3127 (2)	0.0672 (10)	
H22A	0.498045	0.917132	0.260632	0.101*	
H22B	0.495732	0.788375	0.344169	0.101*	
H22C	0.491439	1.017472	0.350196	0.101*	
O7	0.34871 (15)	0.8966 (3)	0.20881 (14)	0.0650 (6)	

### 1,3,5-Trinitrobenzene–1-(naphthalen-2-yl)ethan-1-one (1/1) (I) Atomic displacement parameters (Å^2^)

**Table:**

	*U*^11^	*U*^22^	*U*^33^	*U*^12^	*U*^13^	*U*^23^
C1	0.0476 (19)	0.0333 (14)	0.0369 (15)	−0.0045 (12)	0.0118 (14)	−0.0014 (11)
C2	0.0381 (18)	0.0357 (14)	0.0434 (16)	0.0008 (12)	−0.0011 (14)	−0.0009 (12)
C3	0.0303 (16)	0.0325 (13)	0.0510 (17)	0.0010 (11)	0.0114 (14)	−0.0037 (12)
C4	0.0447 (19)	0.0346 (14)	0.0363 (14)	0.0034 (12)	0.0121 (14)	−0.0028 (11)
C5	0.0352 (17)	0.0306 (13)	0.0421 (15)	0.0039 (11)	−0.0007 (13)	−0.0036 (11)
C6	0.0346 (17)	0.0345 (14)	0.0502 (17)	−0.0023 (12)	0.0121 (14)	−0.0070 (12)
N1	0.073 (2)	0.0471 (15)	0.0475 (16)	−0.0166 (14)	0.0164 (16)	−0.0030 (12)
N2	0.0416 (17)	0.0424 (14)	0.078 (2)	0.0023 (12)	0.0204 (16)	−0.0066 (13)
N3	0.0462 (19)	0.0524 (15)	0.0570 (17)	0.0066 (13)	−0.0114 (14)	−0.0043 (13)
O1	0.095 (2)	0.0840 (17)	0.0698 (16)	−0.0071 (15)	0.0500 (16)	−0.0035 (13)
O2	0.097 (2)	0.0782 (16)	0.0457 (13)	−0.0164 (15)	0.0035 (14)	0.0144 (12)
O3	0.0317 (14)	0.0797 (16)	0.118 (2)	0.0058 (12)	0.0062 (15)	0.0163 (15)
O4	0.0642 (18)	0.0922 (18)	0.0813 (17)	−0.0031 (13)	0.0449 (15)	−0.0105 (14)
O5	0.0794 (19)	0.0802 (16)	0.0436 (12)	0.0271 (13)	−0.0073 (13)	0.0002 (12)
O6	0.0373 (16)	0.101 (2)	0.112 (2)	−0.0084 (14)	−0.0087 (15)	0.0112 (16)
C11	0.0329 (16)	0.0417 (15)	0.0488 (17)	0.0003 (12)	0.0056 (14)	−0.0014 (12)
C12	0.0349 (17)	0.0365 (14)	0.0451 (16)	−0.0017 (12)	0.0102 (13)	−0.0027 (12)
C13	0.0406 (18)	0.0362 (14)	0.0407 (15)	0.0010 (12)	0.0038 (13)	−0.0014 (12)
C14	0.0290 (16)	0.0292 (12)	0.0461 (16)	0.0018 (11)	0.0032 (13)	−0.0040 (11)
C15	0.0361 (18)	0.0404 (15)	0.0538 (17)	0.0053 (12)	0.0010 (15)	−0.0034 (13)
C16	0.0349 (18)	0.0454 (16)	0.068 (2)	0.0061 (13)	0.0131 (16)	−0.0054 (15)
C17	0.044 (2)	0.0430 (16)	0.066 (2)	0.0039 (13)	0.0249 (17)	−0.0054 (14)
C18	0.049 (2)	0.0382 (15)	0.0456 (16)	0.0023 (13)	0.0137 (15)	−0.0019 (12)
C19	0.0359 (17)	0.0307 (14)	0.0413 (15)	0.0020 (11)	0.0063 (13)	−0.0026 (11)
C20	0.0381 (18)	0.0401 (14)	0.0396 (15)	0.0016 (12)	0.0016 (13)	0.0004 (12)
C21	0.052 (2)	0.0463 (16)	0.0466 (18)	0.0000 (14)	0.0180 (16)	−0.0048 (14)
C22	0.046 (2)	0.095 (3)	0.067 (2)	−0.0053 (19)	0.0274 (18)	−0.0026 (19)
O7	0.0648 (16)	0.0863 (16)	0.0466 (13)	−0.0020 (13)	0.0180 (12)	−0.0060 (11)

### 1,3,5-Trinitrobenzene–1-(naphthalen-2-yl)ethan-1-one (1/1) (I) Geometric parameters (Å, º)

**Table:**

C1—C2	1.375 (4)	C12—C13	1.372 (4)
C1—C6	1.375 (4)	C12—C21	1.500 (4)
C1—N1	1.475 (4)	C13—C14	1.416 (4)
C2—C3	1.368 (4)	C13—H13	0.93
C2—H2	0.93	C14—C19	1.415 (4)
C3—C4	1.371 (4)	C14—C15	1.421 (4)
C3—N2	1.481 (4)	C15—C16	1.361 (4)
C4—C5	1.375 (4)	C15—H15	0.93
C4—H4	0.93	C16—C17	1.396 (4)
C5—C6	1.373 (4)	C16—H16	0.93
C5—N3	1.478 (4)	C17—C18	1.372 (4)
C6—H6	0.93	C17—H17	0.93
N1—O2	1.217 (4)	C18—C19	1.418 (4)
N1—O1	1.222 (3)	C18—H18	0.93
N2—O4	1.214 (3)	C19—C20	1.410 (4)
N2—O3	1.224 (3)	C20—H20	0.93
N3—O5	1.212 (3)	C21—O7	1.215 (3)
N3—O6	1.240 (3)	C21—C22	1.497 (4)
C11—C20	1.357 (4)	C22—H22A	0.96
C11—C12	1.422 (4)	C22—H22B	0.96
C11—H11	0.93	C22—H22C	0.96
			
C2—C1—C6	123.4 (2)	C12—C13—C14	121.1 (3)
C2—C1—N1	117.7 (3)	C12—C13—H13	119.5
C6—C1—N1	118.9 (3)	C14—C13—H13	119.5
C3—C2—C1	116.5 (3)	C19—C14—C13	119.2 (2)
C3—C2—H2	121.8	C19—C14—C15	119.3 (2)
C1—C2—H2	121.8	C13—C14—C15	121.5 (2)
C2—C3—C4	123.5 (2)	C16—C15—C14	120.2 (3)
C2—C3—N2	118.1 (3)	C16—C15—H15	119.9
C4—C3—N2	118.4 (2)	C14—C15—H15	119.9
C3—C4—C5	117.0 (2)	C15—C16—C17	120.8 (3)
C3—C4—H4	121.5	C15—C16—H16	119.6
C5—C4—H4	121.5	C17—C16—H16	119.6
C6—C5—C4	122.8 (3)	C18—C17—C16	120.5 (3)
C6—C5—N3	118.1 (3)	C18—C17—H17	119.7
C4—C5—N3	119.0 (2)	C16—C17—H17	119.7
C5—C6—C1	116.8 (3)	C17—C18—C19	120.4 (3)
C5—C6—H6	121.6	C17—C18—H18	119.8
C1—C6—H6	121.6	C19—C18—H18	119.8
O2—N1—O1	125.4 (3)	C20—C19—C14	118.9 (2)
O2—N1—C1	117.0 (3)	C20—C19—C18	122.4 (2)
O1—N1—C1	117.6 (3)	C14—C19—C18	118.7 (3)
O4—N2—O3	125.5 (3)	C11—C20—C19	120.8 (3)
O4—N2—C3	117.2 (3)	C11—C20—H20	119.6
O3—N2—C3	117.3 (3)	C19—C20—H20	119.6
O5—N3—O6	126.0 (3)	O7—C21—C22	121.3 (3)
O5—N3—C5	117.3 (3)	O7—C21—C12	120.2 (3)
O6—N3—C5	116.6 (3)	C22—C21—C12	118.4 (3)
C20—C11—C12	121.1 (3)	C21—C22—H22A	109.5
C20—C11—H11	119.4	C21—C22—H22B	109.5
C12—C11—H11	119.4	H22A—C22—H22B	109.5
C13—C12—C11	118.9 (2)	C21—C22—H22C	109.5
C13—C12—C21	119.2 (3)	H22A—C22—H22C	109.5
C11—C12—C21	121.9 (3)	H22B—C22—H22C	109.5
			
C6—C1—C2—C3	0.9 (4)	C20—C11—C12—C13	1.5 (4)
N1—C1—C2—C3	−179.4 (2)	C20—C11—C12—C21	−177.5 (2)
C1—C2—C3—C4	−0.6 (4)	C11—C12—C13—C14	−0.8 (4)
C1—C2—C3—N2	−179.8 (2)	C21—C12—C13—C14	178.3 (2)
C2—C3—C4—C5	−0.3 (4)	C12—C13—C14—C19	−0.9 (3)
N2—C3—C4—C5	178.9 (2)	C12—C13—C14—C15	−178.7 (2)
C3—C4—C5—C6	0.9 (4)	C19—C14—C15—C16	−0.9 (4)
C3—C4—C5—N3	−179.1 (2)	C13—C14—C15—C16	176.9 (2)
C4—C5—C6—C1	−0.7 (4)	C14—C15—C16—C17	0.7 (4)
N3—C5—C6—C1	179.4 (2)	C15—C16—C17—C18	0.0 (4)
C2—C1—C6—C5	−0.2 (4)	C16—C17—C18—C19	−0.6 (4)
N1—C1—C6—C5	−179.9 (2)	C13—C14—C19—C20	1.8 (3)
C2—C1—N1—O2	−20.0 (4)	C15—C14—C19—C20	179.6 (2)
C6—C1—N1—O2	159.7 (3)	C13—C14—C19—C18	−177.6 (2)
C2—C1—N1—O1	161.3 (3)	C15—C14—C19—C18	0.3 (3)
C6—C1—N1—O1	−19.0 (4)	C17—C18—C19—C20	−178.9 (2)
C2—C3—N2—O4	−175.6 (2)	C17—C18—C19—C14	0.4 (4)
C4—C3—N2—O4	5.2 (4)	C12—C11—C20—C19	−0.6 (4)
C2—C3—N2—O3	4.9 (4)	C14—C19—C20—C11	−1.1 (3)
C4—C3—N2—O3	−174.4 (2)	C18—C19—C20—C11	178.2 (2)
C6—C5—N3—O5	166.6 (2)	C13—C12—C21—O7	−7.3 (4)
C4—C5—N3—O5	−13.4 (3)	C11—C12—C21—O7	171.7 (3)
C6—C5—N3—O6	−10.7 (4)	C13—C12—C21—C22	173.0 (3)
C4—C5—N3—O6	169.3 (3)	C11—C12—C21—C22	−8.0 (4)

### 1,3,5-Trinitrobenzene–1-(naphthalen-2-yl)ethan-1-one (1/1) (I) Hydrogen-bond geometry (Å, º)

**Table:**

*D*—H···*A*	*D*—H	H···*A*	*D*···*A*	*D*—H···*A*
C4—H4···O2^i^	0.93	2.4	3.189 (3)	143
C11—H11···O3^ii^	0.93	2.48	3.323 (4)	150
C22—H22*A*···O4^iii^	0.96	2.64	3.554 (4)	159

Symmetry codes: (i) *x*, −*y*+1/2, *z*−1/2; (ii) −*x*+1, −*y*+1, −*z*+1; (iii) −*x*+1, *y*+1/2, −*z*+1/2.

### 1,3,5-Trinitrobenzene–9-bromoanthracene (1/1) (II) Crystal data

**Table:**

C_14_H_9_Br·C_6_H_3_N_3_O_6_	*F*(000) = 944
*M**_r_* = 470.24	*D*_x_ = 1.695 Mg m^−^^3^
Monoclinic, *P*2_1_/*c*	Mo *K*α radiation, λ = 0.71073 Å
Hall symbol: -P 2ybc	Cell parameters from 8164 reflections
*a* = 7.0928 (2) Å	θ = 2.2–27.9°
*b* = 9.7701 (3) Å	µ = 2.28 mm^−^^1^
*c* = 27.0563 (7) Å	*T* = 173 K
β = 100.674 (1)°	Needle, yellow
*V* = 1842.49 (9) Å^3^	0.33 × 0.12 × 0.11 mm
*Z* = 4	

### 1,3,5-Trinitrobenzene–9-bromoanthracene (1/1) (II) Data collection

**Table:**

Bruker D8 Venture Photon CCD area detector diffractometer	3758 reflections with *I* > 2σ(*I*)
Graphite monochromator	*R*_int_ = 0.048
ω scans	θ_max_ = 28.0°, θ_min_ = 1.5°
Absorption correction: multi-scan (SADABS; Sheldrick, 1996)	*h* = −9→9
*T*_min_ = 0.56, *T*_max_ = 0.77	*k* = −12→12
28424 measured reflections	*l* = −35→35
4450 independent reflections	

### 1,3,5-Trinitrobenzene–9-bromoanthracene (1/1) (II) Refinement

**Table:**

Refinement on *F*^2^	0 constraints
Least-squares matrix: full	Hydrogen site location: inferred from neighbouring sites
*R*[*F*^2^ > 2σ(*F*^2^)] = 0.040	H-atom parameters constrained
*wR*(*F*^2^) = 0.096	*w* = 1/[σ^2^(*F*_o_^2^) + (0.0343*P*)^2^ + 2.1935*P*] where *P* = (*F*_o_^2^ + 2*F*_c_^2^)/3
*S* = 1.08	(Δ/σ)_max_ < 0.001
4450 reflections	Δρ_max_ = 1.42 e Å^−^^3^
271 parameters	Δρ_min_ = −0.94 e Å^−^^3^
0 restraints	

### 1,3,5-Trinitrobenzene–9-bromoanthracene (1/1) (II) Special details

**Table:**

Experimental. (SADABS; Sheldrick, 1996)
Geometry. All standard uncertainties (s.u.) (except the s.u. in the dihedral angle between two l.s. planes) are estimated using the full covariance matrix. The cell s.u. are taken into account individually in the estimation of s.u. in distances, angles and torsion angles; correlations between s.u. in cell parameters are only used when they are defined by crystal symmetry. An approximate (isotropic) treatment of cell s.u. is used for estimating s.u. involving l.s. planes.

### 1,3,5-Trinitrobenzene–9-bromoanthracene (1/1) (II) Fractional atomic coordinates and isotropic or equivalent isotropic displacement parameters (Å^2^)

**Table:**

	*x*	*y*	*z*	*U*_iso_*/*U*_eq_	
C1	0.3620 (3)	0.7554 (2)	0.10545 (9)	0.0275 (5)	
C2	0.3965 (3)	0.6350 (2)	0.08161 (9)	0.0256 (5)	
H2	0.369113	0.62674	0.046011	0.031*	
C3	0.4723 (3)	0.5280 (2)	0.11194 (9)	0.0236 (4)	
C4	0.5164 (3)	0.5366 (2)	0.16372 (9)	0.0267 (5)	
H4	0.568061	0.460884	0.183771	0.032*	
C5	0.4822 (4)	0.6599 (3)	0.18498 (9)	0.0293 (5)	
C6	0.4047 (4)	0.7724 (2)	0.15702 (10)	0.0303 (5)	
H6	0.382248	0.856585	0.172517	0.036*	
N1	0.2782 (3)	0.8716 (2)	0.07398 (10)	0.0370 (5)	
N2	0.5097 (3)	0.3967 (2)	0.08822 (8)	0.0290 (4)	
N3	0.5319 (4)	0.6732 (2)	0.24030 (8)	0.0401 (6)	
O1	0.2533 (3)	0.9777 (2)	0.09572 (9)	0.0543 (6)	
O2	0.2376 (3)	0.8538 (2)	0.02904 (9)	0.0519 (6)	
O3	0.4729 (3)	0.3904 (2)	0.04231 (7)	0.0396 (4)	
O4	0.5756 (3)	0.30406 (19)	0.11577 (8)	0.0434 (5)	
O5	0.5774 (5)	0.5714 (2)	0.26496 (8)	0.0654 (7)	
O6	0.5271 (4)	0.7868 (3)	0.25776 (9)	0.0634 (7)	
C11	0.9660 (3)	0.6511 (2)	0.16436 (8)	0.0235 (4)	
C12	0.8864 (3)	0.7691 (2)	0.13900 (8)	0.0231 (4)	
C13	0.8514 (4)	0.8940 (2)	0.16310 (9)	0.0305 (5)	
H13	0.883923	0.900938	0.198681	0.037*	
C14	0.7719 (4)	1.0031 (2)	0.13575 (10)	0.0338 (6)	
H14	0.748685	1.084959	0.15261	0.041*	
C15	0.7233 (4)	0.9972 (3)	0.08274 (10)	0.0319 (5)	
H15	0.668981	1.074876	0.064272	0.038*	
C16	0.7543 (3)	0.8804 (2)	0.05821 (9)	0.0285 (5)	
H16	0.721939	0.877421	0.02255	0.034*	
C17	0.8346 (3)	0.7622 (2)	0.08509 (8)	0.0223 (4)	
C18	0.8607 (3)	0.6406 (2)	0.06047 (8)	0.0239 (5)	
H18	0.824385	0.636703	0.024888	0.029*	
C19	0.9385 (3)	0.5241 (2)	0.08641 (8)	0.0226 (4)	
C20	0.9636 (3)	0.4002 (2)	0.06075 (9)	0.0274 (5)	
H20	0.924336	0.396003	0.02525	0.033*	
C21	1.0428 (4)	0.2876 (3)	0.08608 (10)	0.0322 (5)	
H21	1.058174	0.205658	0.068314	0.039*	
C22	1.1019 (4)	0.2928 (3)	0.13873 (10)	0.0319 (5)	
H22	1.159053	0.214283	0.156067	0.038*	
C23	1.0789 (3)	0.4077 (2)	0.16519 (9)	0.0282 (5)	
H23	1.118668	0.408027	0.200702	0.034*	
C24	0.9956 (3)	0.5283 (2)	0.14027 (8)	0.0224 (4)	
Br1	1.03399 (4)	0.65915 (3)	0.23592 (2)	0.03853 (10)	

### 1,3,5-Trinitrobenzene–9-bromoanthracene (1/1) (II) Atomic displacement parameters (Å^2^)

**Table:**

	*U*^11^	*U*^22^	*U*^33^	*U*^12^	*U*^13^	*U*^23^
C1	0.0215 (11)	0.0233 (11)	0.0390 (13)	0.0012 (9)	0.0088 (10)	0.0050 (10)
C2	0.0224 (11)	0.0255 (12)	0.0296 (11)	−0.0013 (9)	0.0067 (9)	0.0014 (9)
C3	0.0203 (10)	0.0211 (11)	0.0306 (11)	−0.0022 (8)	0.0075 (9)	−0.0027 (9)
C4	0.0268 (12)	0.0232 (11)	0.0307 (12)	−0.0006 (9)	0.0068 (9)	0.0009 (9)
C5	0.0297 (12)	0.0287 (12)	0.0301 (12)	−0.0045 (10)	0.0071 (10)	−0.0041 (10)
C6	0.0309 (13)	0.0228 (12)	0.0393 (13)	−0.0013 (10)	0.0121 (10)	−0.0064 (10)
N1	0.0296 (11)	0.0304 (12)	0.0515 (14)	0.0040 (9)	0.0094 (10)	0.0117 (10)
N2	0.0264 (10)	0.0259 (10)	0.0353 (11)	−0.0005 (8)	0.0076 (8)	−0.0060 (9)
N3	0.0492 (14)	0.0377 (13)	0.0331 (12)	−0.0039 (11)	0.0068 (10)	−0.0095 (10)
O1	0.0568 (14)	0.0288 (11)	0.0759 (16)	0.0161 (10)	0.0087 (12)	0.0048 (10)
O2	0.0588 (14)	0.0489 (13)	0.0476 (13)	0.0129 (11)	0.0091 (10)	0.0208 (10)
O3	0.0412 (11)	0.0416 (11)	0.0347 (10)	0.0031 (9)	0.0040 (8)	−0.0129 (8)
O4	0.0580 (13)	0.0250 (9)	0.0485 (12)	0.0132 (9)	0.0136 (10)	0.0009 (8)
O5	0.116 (2)	0.0441 (13)	0.0327 (11)	−0.0039 (13)	0.0041 (12)	0.0036 (10)
O6	0.0894 (19)	0.0487 (13)	0.0455 (13)	0.0123 (13)	−0.0048 (12)	−0.0221 (11)
C11	0.0252 (11)	0.0260 (11)	0.0195 (10)	0.0006 (9)	0.0044 (8)	0.0025 (8)
C12	0.0239 (11)	0.0214 (11)	0.0242 (11)	0.0001 (9)	0.0047 (9)	0.0014 (8)
C13	0.0406 (14)	0.0230 (11)	0.0279 (12)	0.0002 (10)	0.0061 (10)	−0.0032 (9)
C14	0.0423 (15)	0.0183 (11)	0.0409 (14)	0.0006 (10)	0.0077 (11)	−0.0026 (10)
C15	0.0334 (13)	0.0232 (12)	0.0388 (13)	0.0023 (10)	0.0062 (10)	0.0078 (10)
C16	0.0297 (12)	0.0277 (12)	0.0275 (11)	0.0021 (10)	0.0042 (9)	0.0061 (9)
C17	0.0199 (10)	0.0233 (11)	0.0245 (10)	−0.0006 (8)	0.0060 (8)	0.0031 (8)
C18	0.0225 (11)	0.0290 (12)	0.0207 (10)	0.0019 (9)	0.0053 (8)	0.0003 (8)
C19	0.0188 (10)	0.0242 (11)	0.0260 (11)	0.0005 (8)	0.0071 (8)	−0.0003 (9)
C20	0.0249 (11)	0.0286 (12)	0.0300 (12)	−0.0002 (9)	0.0086 (9)	−0.0047 (10)
C21	0.0301 (13)	0.0227 (11)	0.0465 (15)	0.0021 (10)	0.0146 (11)	−0.0055 (10)
C22	0.0309 (13)	0.0219 (11)	0.0443 (14)	0.0046 (10)	0.0103 (11)	0.0073 (10)
C23	0.0274 (12)	0.0252 (12)	0.0319 (12)	0.0020 (9)	0.0056 (10)	0.0057 (9)
C24	0.0197 (10)	0.0216 (11)	0.0267 (11)	0.0000 (8)	0.0061 (8)	0.0019 (8)
Br1	0.05475 (19)	0.03823 (16)	0.02111 (13)	0.01206 (12)	0.00313 (10)	0.00126 (10)

### 1,3,5-Trinitrobenzene–9-bromoanthracene (1/1) (II) Geometric parameters (Å, º)

**Table:**

C1—C6	1.382 (4)	C12—C17	1.438 (3)
C1—C2	1.385 (3)	C13—C14	1.359 (3)
C1—N1	1.477 (3)	C13—H13	0.95
C2—C3	1.376 (3)	C14—C15	1.413 (4)
C2—H2	0.95	C14—H14	0.95
C3—C4	1.381 (3)	C15—C16	1.358 (4)
C3—N2	1.480 (3)	C15—H15	0.95
C4—C5	1.376 (3)	C16—C17	1.426 (3)
C4—H4	0.95	C16—H16	0.95
C5—C6	1.388 (3)	C17—C18	1.391 (3)
C5—N3	1.479 (3)	C18—C19	1.396 (3)
C6—H6	0.95	C18—H18	0.95
N1—O2	1.209 (3)	C19—C20	1.423 (3)
N1—O1	1.221 (3)	C19—C24	1.439 (3)
N2—O4	1.210 (3)	C20—C21	1.362 (4)
N2—O3	1.222 (3)	C20—H20	0.95
N3—O5	1.208 (3)	C21—C22	1.409 (4)
N3—O6	1.209 (3)	C21—H21	0.95
C11—C24	1.400 (3)	C22—C23	1.358 (4)
C11—C12	1.405 (3)	C22—H22	0.95
C11—Br1	1.908 (2)	C23—C24	1.430 (3)
C12—C13	1.426 (3)	C23—H23	0.95
			
C6—C1—C2	123.2 (2)	C12—C13—H13	119.6
C6—C1—N1	118.6 (2)	C13—C14—C15	121.3 (2)
C2—C1—N1	118.1 (2)	C13—C14—H14	119.4
C3—C2—C1	116.8 (2)	C15—C14—H14	119.4
C3—C2—H2	121.6	C16—C15—C14	119.9 (2)
C1—C2—H2	121.6	C16—C15—H15	120
C2—C3—C4	123.3 (2)	C14—C15—H15	120
C2—C3—N2	118.8 (2)	C15—C16—C17	121.1 (2)
C4—C3—N2	117.9 (2)	C15—C16—H16	119.4
C5—C4—C3	117.0 (2)	C17—C16—H16	119.4
C5—C4—H4	121.5	C18—C17—C16	121.6 (2)
C3—C4—H4	121.5	C18—C17—C12	119.6 (2)
C4—C5—C6	123.2 (2)	C16—C17—C12	118.8 (2)
C4—C5—N3	118.2 (2)	C17—C18—C19	122.1 (2)
C6—C5—N3	118.6 (2)	C17—C18—H18	119
C1—C6—C5	116.4 (2)	C19—C18—H18	119
C1—C6—H6	121.8	C18—C19—C20	121.5 (2)
C5—C6—H6	121.8	C18—C19—C24	119.8 (2)
O2—N1—O1	125.3 (2)	C20—C19—C24	118.8 (2)
O2—N1—C1	117.7 (2)	C21—C20—C19	121.3 (2)
O1—N1—C1	117.0 (2)	C21—C20—H20	119.4
O4—N2—O3	125.0 (2)	C19—C20—H20	119.4
O4—N2—C3	117.4 (2)	C20—C21—C22	119.8 (2)
O3—N2—C3	117.6 (2)	C20—C21—H21	120.1
O5—N3—O6	124.3 (2)	C22—C21—H21	120.1
O5—N3—C5	118.4 (2)	C23—C22—C21	121.4 (2)
O6—N3—C5	117.2 (2)	C23—C22—H22	119.3
C24—C11—C12	123.9 (2)	C21—C22—H22	119.3
C24—C11—Br1	118.43 (16)	C22—C23—C24	120.8 (2)
C12—C11—Br1	117.69 (17)	C22—C23—H23	119.6
C11—C12—C13	124.5 (2)	C24—C23—H23	119.6
C11—C12—C17	117.4 (2)	C11—C24—C23	124.9 (2)
C13—C12—C17	118.0 (2)	C11—C24—C19	117.23 (19)
C14—C13—C12	120.8 (2)	C23—C24—C19	117.9 (2)
C14—C13—H13	119.6		
			
C6—C1—C2—C3	1.8 (4)	C17—C12—C13—C14	−0.2 (4)
N1—C1—C2—C3	−179.2 (2)	C12—C13—C14—C15	−0.6 (4)
C1—C2—C3—C4	−0.9 (3)	C13—C14—C15—C16	0.6 (4)
C1—C2—C3—N2	179.4 (2)	C14—C15—C16—C17	0.4 (4)
C2—C3—C4—C5	−0.3 (4)	C15—C16—C17—C18	177.7 (2)
N2—C3—C4—C5	179.4 (2)	C15—C16—C17—C12	−1.2 (4)
C3—C4—C5—C6	0.8 (4)	C11—C12—C17—C18	1.3 (3)
C3—C4—C5—N3	−178.7 (2)	C13—C12—C17—C18	−177.8 (2)
C2—C1—C6—C5	−1.3 (4)	C11—C12—C17—C16	−179.7 (2)
N1—C1—C6—C5	179.6 (2)	C13—C12—C17—C16	1.2 (3)
C4—C5—C6—C1	0.0 (4)	C16—C17—C18—C19	−179.8 (2)
N3—C5—C6—C1	179.5 (2)	C12—C17—C18—C19	−0.8 (3)
C6—C1—N1—O2	−178.3 (2)	C17—C18—C19—C20	179.7 (2)
C2—C1—N1—O2	2.6 (3)	C17—C18—C19—C24	−0.4 (3)
C6—C1—N1—O1	0.8 (3)	C18—C19—C20—C21	178.8 (2)
C2—C1—N1—O1	−178.3 (2)	C24—C19—C20—C21	−1.0 (3)
C2—C3—N2—O4	−179.7 (2)	C19—C20—C21—C22	−0.1 (4)
C4—C3—N2—O4	0.6 (3)	C20—C21—C22—C23	1.1 (4)
C2—C3—N2—O3	0.8 (3)	C21—C22—C23—C24	−0.8 (4)
C4—C3—N2—O3	−179.0 (2)	C12—C11—C24—C23	179.0 (2)
C4—C5—N3—O5	−9.2 (4)	Br1—C11—C24—C23	−1.9 (3)
C6—C5—N3—O5	171.2 (3)	C12—C11—C24—C19	−0.7 (3)
C4—C5—N3—O6	169.6 (3)	Br1—C11—C24—C19	178.46 (16)
C6—C5—N3—O6	−9.9 (4)	C22—C23—C24—C11	179.9 (2)
C24—C11—C12—C13	178.5 (2)	C22—C23—C24—C19	−0.5 (3)
Br1—C11—C12—C13	−0.6 (3)	C18—C19—C24—C11	1.2 (3)
C24—C11—C12—C17	−0.6 (3)	C20—C19—C24—C11	−179.0 (2)
Br1—C11—C12—C17	−179.70 (16)	C18—C19—C24—C23	−178.5 (2)
C11—C12—C13—C14	−179.3 (2)	C20—C19—C24—C23	1.3 (3)

### 1,3,5-Trinitrobenzene–9-bromoanthracene (1/1) (II) Hydrogen-bond geometry (Å, º)

**Table:**

*D*—H···*A*	*D*—H	H···*A*	*D*···*A*	*D*—H···*A*
C4—H4···O6^i^	0.95	2.5	3.287 (3)	140
C6—H6···O5^ii^	0.95	2.68	3.593 (3)	162
C14—H14···O4^iii^	0.95	2.57	3.255 (3)	129
C21—H21···O1^iv^	0.95	2.65	3.364 (3)	132

Symmetry codes: (i) −*x*+1, *y*−1/2, −*z*+1/2; (ii) −*x*+1, *y*+1/2, −*z*+1/2; (iii) *x*, *y*+1, *z*; (iv) *x*+1, *y*−1, *z*.

### 1,3,5-Trinitrobenzene–2-{(E)-[4-(dimethylamino)phenyl]diazenyl}benzoic acid (1/1) (III) Crystal data

**Table:**

C_15_H_15_N_3_O_2_·C_6_H_3_N_3_O_6_	*Z* = 2
*M**_r_* = 482.41	*F*(000) = 500
Triclinic, *P*1	*D*_x_ = 1.518 Mg m^−^^3^
Hall symbol: -P 1	Mo *K*α radiation, λ = 0.71073 Å
*a* = 8.550 (3) Å	Cell parameters from 9824 reflections
*b* = 10.437 (3) Å	θ = 3.1–28.2°
*c* = 13.072 (5) Å	µ = 0.12 mm^−^^1^
α = 110.689 (10)°	*T* = 173 K
β = 103.510 (12)°	Cuboid, red
γ = 90.730 (12)°	0.29 × 0.13 × 0.12 mm
*V* = 1055.2 (7) Å^3^	

### 1,3,5-Trinitrobenzene–2-{(E)-[4-(dimethylamino)phenyl]diazenyl}benzoic acid (1/1) (III) Data collection

**Table:**

Bruker D8 Venture Photon CCD area detector diffractometer	3262 reflections with *I* > 2σ(*I*)
Graphite monochromator	*R*_int_ = 0.072
ω scans	θ_max_ = 28.0°, θ_min_ = 3.4°
Absorption correction: multi-scan (SADABS; Sheldrick, 1996)	*h* = −11→11
*T*_min_ = 0.9, *T*_max_ = 0.95	*k* = −13→13
39527 measured reflections	*l* = −17→17
5072 independent reflections	

### 1,3,5-Trinitrobenzene–2-{(E)-[4-(dimethylamino)phenyl]diazenyl}benzoic acid (1/1) (III) Refinement

**Table:**

Refinement on *F*^2^	0 constraints
Least-squares matrix: full	Hydrogen site location: mixed
*R*[*F*^2^ > 2σ(*F*^2^)] = 0.078	H atoms treated by a mixture of independent and constrained refinement
*wR*(*F*^2^) = 0.249	*w* = 1/[σ^2^(*F*_o_^2^) + (0.1346*P*)^2^ + 0.8758*P*] where *P* = (*F*_o_^2^ + 2*F*_c_^2^)/3
*S* = 1.07	(Δ/σ)_max_ < 0.001
5072 reflections	Δρ_max_ = 0.49 e Å^−^^3^
322 parameters	Δρ_min_ = −0.43 e Å^−^^3^
0 restraints	

### 1,3,5-Trinitrobenzene–2-{(E)-[4-(dimethylamino)phenyl]diazenyl}benzoic acid (1/1) (III) Special details

**Table:**

Experimental. (SADABS; Sheldrick, 1996)
Geometry. All standard uncertainties (s.u.) (except the s.u. in the dihedral angle between two l.s. planes) are estimated using the full covariance matrix. The cell s.u. are taken into account individually in the estimation of s.u. in distances, angles and torsion angles; correlations between s.u. in cell parameters are only used when they are defined by crystal symmetry. An approximate (isotropic) treatment of cell s.u. is used for estimating s.u. involving l.s. planes.

### 1,3,5-Trinitrobenzene–2-{(E)-[4-(dimethylamino)phenyl]diazenyl}benzoic acid (1/1) (III) Fractional atomic coordinates and isotropic or equivalent isotropic displacement parameters (Å^2^)

**Table:**

	*x*	*y*	*z*	*U*_iso_*/*U*_eq_	
C1	0.3627 (3)	0.4196 (3)	0.8376 (2)	0.0274 (6)	
C2	0.4468 (3)	0.3469 (3)	0.7610 (2)	0.0265 (6)	
H2	0.49673	0.267951	0.76649	0.032*	
C3	0.4549 (3)	0.3944 (3)	0.6758 (2)	0.0266 (6)	
C4	0.3817 (3)	0.5079 (3)	0.6651 (2)	0.0279 (6)	
H4	0.386476	0.537651	0.605026	0.033*	
C5	0.3010 (4)	0.5763 (3)	0.7456 (3)	0.0287 (6)	
C6	0.2887 (3)	0.5341 (3)	0.8327 (2)	0.0284 (6)	
H6	0.231723	0.582036	0.88668	0.034*	
N1	0.3560 (3)	0.3737 (3)	0.9312 (2)	0.0324 (6)	
N2	0.5442 (3)	0.3204 (3)	0.5938 (2)	0.0305 (6)	
N3	0.2291 (3)	0.7015 (3)	0.7399 (2)	0.0367 (6)	
O1	0.2796 (3)	0.4374 (3)	0.9979 (2)	0.0475 (6)	
O2	0.4303 (3)	0.2766 (3)	0.93921 (19)	0.0437 (6)	
O3	0.6018 (3)	0.2169 (2)	0.6015 (2)	0.0405 (6)	
O4	0.5548 (3)	0.3668 (3)	0.5212 (2)	0.0435 (6)	
O5	0.2266 (3)	0.7301 (3)	0.6567 (2)	0.0485 (7)	
O6	0.1772 (3)	0.7707 (3)	0.8201 (2)	0.0507 (7)	
C11	0.1578 (3)	0.1335 (3)	0.5551 (2)	0.0255 (6)	
C12	0.1935 (4)	0.0433 (3)	0.6139 (2)	0.0272 (6)	
H12A	0.258146	−0.028852	0.588579	0.033*	
C13	0.1372 (3)	0.0571 (3)	0.7067 (2)	0.0278 (6)	
H13	0.163717	−0.004867	0.745181	0.033*	
C14	0.0402 (3)	0.1627 (3)	0.7454 (2)	0.0262 (6)	
C15	−0.0005 (3)	0.2516 (3)	0.6840 (3)	0.0293 (6)	
H15	−0.068293	0.32188	0.707451	0.035*	
C16	0.0572 (3)	0.2365 (3)	0.5914 (3)	0.0283 (6)	
H16	0.028785	0.296526	0.551294	0.034*	
C17	0.0277 (4)	0.0891 (4)	0.9028 (3)	0.0388 (8)	
H17A	−0.012745	−0.006156	0.852959	0.058*	
H17B	−0.022233	0.116076	0.966614	0.058*	
H17C	0.145383	0.096273	0.93118	0.058*	
C18	−0.1074 (5)	0.2903 (4)	0.8855 (3)	0.0444 (9)	
H18A	−0.116874	0.351673	0.842831	0.067*	
H18B	−0.053611	0.342523	0.965088	0.067*	
H18C	−0.215479	0.251383	0.87965	0.067*	
C19	0.2781 (3)	0.1679 (3)	0.3235 (2)	0.0265 (6)	
C20	0.3656 (4)	0.0550 (3)	0.2908 (3)	0.0336 (7)	
H20	0.380119	−0.005691	0.331185	0.04*	
C21	0.4310 (4)	0.0317 (3)	0.1995 (3)	0.0378 (7)	
H21	0.490257	−0.045347	0.177299	0.045*	
C22	0.4111 (4)	0.1198 (3)	0.1398 (3)	0.0347 (7)	
H22	0.455378	0.10206	0.076507	0.042*	
C23	0.3273 (4)	0.2324 (3)	0.1724 (2)	0.0315 (7)	
H23	0.314185	0.292407	0.131361	0.038*	
C24	0.2613 (3)	0.2597 (3)	0.2650 (2)	0.0259 (6)	
C25	0.1757 (4)	0.3865 (3)	0.2948 (3)	0.0338 (7)	
N11	0.2263 (3)	0.1105 (2)	0.4652 (2)	0.0280 (6)	
N12	0.2057 (3)	0.1977 (3)	0.4154 (2)	0.0282 (6)	
N13	−0.0133 (3)	0.1802 (3)	0.8396 (2)	0.0343 (6)	
O11	0.1681 (4)	0.4628 (3)	0.2426 (2)	0.0520 (7)	
O12	0.1077 (3)	0.4150 (2)	0.3810 (2)	0.0395 (6)	
H12	0.130 (6)	0.347 (6)	0.413 (5)	0.096 (18)*	

### 1,3,5-Trinitrobenzene–2-{(E)-[4-(dimethylamino)phenyl]diazenyl}benzoic acid (1/1) (III) Atomic displacement parameters (Å^2^)

**Table:**

	*U*^11^	*U*^22^	*U*^33^	*U*^12^	*U*^13^	*U*^23^
C1	0.0319 (15)	0.0312 (16)	0.0233 (14)	−0.0012 (12)	0.0082 (12)	0.0144 (12)
C2	0.0295 (15)	0.0266 (15)	0.0271 (14)	0.0022 (11)	0.0080 (12)	0.0138 (11)
C3	0.0283 (15)	0.0286 (15)	0.0275 (14)	0.0014 (11)	0.0117 (12)	0.0130 (12)
C4	0.0310 (15)	0.0302 (16)	0.0273 (14)	0.0010 (12)	0.0080 (12)	0.0162 (12)
C5	0.0340 (16)	0.0247 (15)	0.0322 (15)	0.0045 (12)	0.0108 (12)	0.0147 (12)
C6	0.0308 (15)	0.0286 (16)	0.0284 (15)	0.0018 (12)	0.0111 (12)	0.0115 (12)
N1	0.0394 (15)	0.0364 (15)	0.0263 (13)	0.0010 (11)	0.0120 (11)	0.0150 (11)
N2	0.0340 (14)	0.0340 (14)	0.0290 (13)	0.0028 (11)	0.0128 (11)	0.0151 (11)
N3	0.0400 (15)	0.0311 (15)	0.0463 (16)	0.0088 (11)	0.0155 (13)	0.0197 (12)
O1	0.0657 (17)	0.0558 (16)	0.0391 (13)	0.0174 (12)	0.0322 (12)	0.0264 (11)
O2	0.0643 (16)	0.0442 (14)	0.0379 (13)	0.0172 (12)	0.0205 (12)	0.0283 (11)
O3	0.0502 (14)	0.0396 (13)	0.0461 (14)	0.0167 (11)	0.0266 (11)	0.0232 (11)
O4	0.0583 (16)	0.0514 (15)	0.0398 (13)	0.0125 (11)	0.0292 (12)	0.0285 (11)
O5	0.0607 (16)	0.0482 (15)	0.0630 (17)	0.0212 (12)	0.0295 (13)	0.0424 (13)
O6	0.0681 (18)	0.0384 (14)	0.0545 (16)	0.0222 (12)	0.0277 (13)	0.0194 (12)
C11	0.0235 (14)	0.0251 (14)	0.0304 (15)	0.0009 (11)	0.0062 (11)	0.0138 (11)
C12	0.0304 (15)	0.0251 (15)	0.0286 (15)	0.0056 (11)	0.0074 (12)	0.0129 (11)
C13	0.0329 (16)	0.0257 (15)	0.0289 (15)	0.0065 (12)	0.0069 (12)	0.0154 (12)
C14	0.0245 (14)	0.0278 (15)	0.0284 (14)	−0.0001 (11)	0.0056 (11)	0.0134 (12)
C15	0.0269 (15)	0.0301 (16)	0.0361 (16)	0.0075 (12)	0.0095 (12)	0.0173 (13)
C16	0.0280 (15)	0.0289 (15)	0.0332 (15)	0.0029 (11)	0.0062 (12)	0.0185 (12)
C17	0.049 (2)	0.0442 (19)	0.0345 (17)	0.0110 (15)	0.0156 (15)	0.0247 (15)
C18	0.054 (2)	0.047 (2)	0.048 (2)	0.0202 (16)	0.0299 (17)	0.0257 (16)
C19	0.0272 (15)	0.0297 (15)	0.0255 (14)	0.0022 (11)	0.0074 (11)	0.0130 (11)
C20	0.0400 (18)	0.0306 (16)	0.0373 (17)	0.0089 (13)	0.0128 (14)	0.0188 (13)
C21	0.0437 (19)	0.0353 (18)	0.0395 (18)	0.0131 (14)	0.0151 (15)	0.0163 (14)
C22	0.0404 (18)	0.0392 (18)	0.0294 (15)	0.0066 (14)	0.0161 (13)	0.0135 (13)
C23	0.0387 (17)	0.0324 (17)	0.0285 (15)	0.0046 (13)	0.0106 (13)	0.0159 (12)
C24	0.0310 (15)	0.0255 (15)	0.0236 (14)	0.0031 (11)	0.0086 (11)	0.0110 (11)
C25	0.0413 (18)	0.0328 (17)	0.0356 (16)	0.0088 (13)	0.0156 (14)	0.0186 (13)
N11	0.0310 (13)	0.0289 (13)	0.0284 (12)	0.0023 (10)	0.0077 (10)	0.0155 (10)
N12	0.0303 (13)	0.0280 (13)	0.0320 (13)	0.0039 (10)	0.0104 (10)	0.0162 (10)
N13	0.0427 (15)	0.0367 (15)	0.0351 (14)	0.0125 (12)	0.0185 (12)	0.0214 (12)
O11	0.0814 (19)	0.0462 (15)	0.0579 (16)	0.0309 (13)	0.0425 (14)	0.0375 (13)
O12	0.0535 (15)	0.0356 (13)	0.0446 (14)	0.0169 (10)	0.0292 (11)	0.0218 (11)

### 1,3,5-Trinitrobenzene–2-{(E)-[4-(dimethylamino)phenyl]diazenyl}benzoic acid (1/1) (III) Geometric parameters (Å, º)

**Table:**

C1—C6	1.374 (4)	C15—C16	1.371 (4)
C1—C2	1.382 (4)	C15—H15	0.95
C1—N1	1.476 (4)	C16—H16	0.95
C2—C3	1.384 (4)	C17—N13	1.461 (4)
C2—H2	0.95	C17—H17A	0.98
C3—C4	1.382 (4)	C17—H17B	0.98
C3—N2	1.463 (4)	C17—H17C	0.98
C4—C5	1.383 (4)	C18—N13	1.446 (4)
C4—H4	0.95	C18—H18A	0.98
C5—C6	1.383 (4)	C18—H18B	0.98
C5—N3	1.469 (4)	C18—H18C	0.98
C6—H6	0.95	C19—C20	1.396 (4)
N1—O1	1.223 (4)	C19—C24	1.412 (4)
N1—O2	1.225 (4)	C19—N12	1.418 (4)
N2—O3	1.219 (3)	C20—C21	1.380 (5)
N2—O4	1.227 (3)	C20—H20	0.95
N3—O5	1.221 (4)	C21—C22	1.390 (5)
N3—O6	1.228 (4)	C21—H21	0.95
C11—N11	1.381 (4)	C22—C23	1.374 (4)
C11—C16	1.406 (4)	C22—H22	0.95
C11—C12	1.406 (4)	C23—C24	1.395 (4)
C12—C13	1.370 (4)	C23—H23	0.95
C12—H12A	0.95	C24—C25	1.494 (4)
C13—C14	1.408 (4)	C25—O11	1.212 (4)
C13—H13	0.95	C25—O12	1.331 (4)
C14—N13	1.366 (4)	N11—N12	1.285 (3)
C14—C15	1.425 (4)	O12—H12	0.95 (6)
			
C6—C1—C2	123.5 (3)	C15—C16—C11	120.9 (3)
C6—C1—N1	118.4 (3)	C15—C16—H16	119.6
C2—C1—N1	118.1 (3)	C11—C16—H16	119.6
C1—C2—C3	116.9 (3)	N13—C17—H17A	109.5
C1—C2—H2	121.5	N13—C17—H17B	109.5
C3—C2—H2	121.5	H17A—C17—H17B	109.5
C4—C3—C2	122.6 (3)	N13—C17—H17C	109.5
C4—C3—N2	119.1 (2)	H17A—C17—H17C	109.5
C2—C3—N2	118.3 (3)	H17B—C17—H17C	109.5
C3—C4—C5	117.2 (3)	N13—C18—H18A	109.5
C3—C4—H4	121.4	N13—C18—H18B	109.5
C5—C4—H4	121.4	H18A—C18—H18B	109.5
C6—C5—C4	122.9 (3)	N13—C18—H18C	109.5
C6—C5—N3	118.6 (3)	H18A—C18—H18C	109.5
C4—C5—N3	118.5 (3)	H18B—C18—H18C	109.5
C1—C6—C5	116.9 (3)	C20—C19—C24	119.7 (3)
C1—C6—H6	121.6	C20—C19—N12	123.1 (3)
C5—C6—H6	121.6	C24—C19—N12	117.3 (2)
O1—N1—O2	124.0 (3)	C21—C20—C19	119.8 (3)
O1—N1—C1	117.8 (3)	C21—C20—H20	120.1
O2—N1—C1	118.2 (3)	C19—C20—H20	120.1
O3—N2—O4	124.5 (3)	C20—C21—C22	120.8 (3)
O3—N2—C3	118.1 (2)	C20—C21—H21	119.6
O4—N2—C3	117.3 (3)	C22—C21—H21	119.6
O5—N3—O6	124.7 (3)	C23—C22—C21	120.0 (3)
O5—N3—C5	117.9 (3)	C23—C22—H22	120
O6—N3—C5	117.3 (3)	C21—C22—H22	120
N11—C11—C16	126.2 (3)	C22—C23—C24	120.7 (3)
N11—C11—C12	115.5 (3)	C22—C23—H23	119.7
C16—C11—C12	118.3 (3)	C24—C23—H23	119.7
C13—C12—C11	121.6 (3)	C23—C24—C19	119.1 (3)
C13—C12—H12A	119.2	C23—C24—C25	116.4 (3)
C11—C12—H12A	119.2	C19—C24—C25	124.5 (3)
C12—C13—C14	120.3 (3)	O11—C25—O12	120.1 (3)
C12—C13—H13	119.9	O11—C25—C24	121.4 (3)
C14—C13—H13	119.9	O12—C25—C24	118.4 (3)
N13—C14—C13	121.1 (3)	N12—N11—C11	116.7 (2)
N13—C14—C15	120.6 (3)	N11—N12—C19	114.0 (2)
C13—C14—C15	118.3 (3)	C14—N13—C18	123.2 (3)
C16—C15—C14	120.6 (3)	C14—N13—C17	120.9 (3)
C16—C15—H15	119.7	C18—N13—C17	115.9 (3)
C14—C15—H15	119.7	C25—O12—H12	108 (3)
			
C6—C1—C2—C3	0.0 (4)	N13—C14—C15—C16	178.1 (3)
N1—C1—C2—C3	−178.3 (3)	C13—C14—C15—C16	−1.7 (4)
C1—C2—C3—C4	−0.8 (4)	C14—C15—C16—C11	−0.1 (4)
C1—C2—C3—N2	179.5 (2)	N11—C11—C16—C15	−178.3 (3)
C2—C3—C4—C5	1.5 (4)	C12—C11—C16—C15	2.1 (4)
N2—C3—C4—C5	−178.9 (3)	C24—C19—C20—C21	−1.8 (5)
C3—C4—C5—C6	−1.4 (4)	N12—C19—C20—C21	179.2 (3)
C3—C4—C5—N3	176.9 (3)	C19—C20—C21—C22	0.1 (5)
C2—C1—C6—C5	0.0 (4)	C20—C21—C22—C23	0.8 (5)
N1—C1—C6—C5	178.4 (3)	C21—C22—C23—C24	−0.1 (5)
C4—C5—C6—C1	0.7 (4)	C22—C23—C24—C19	−1.6 (4)
N3—C5—C6—C1	−177.6 (3)	C22—C23—C24—C25	178.7 (3)
C6—C1—N1—O1	2.5 (4)	C20—C19—C24—C23	2.5 (4)
C2—C1—N1—O1	−179.1 (3)	N12—C19—C24—C23	−178.5 (3)
C6—C1—N1—O2	−175.7 (3)	C20—C19—C24—C25	−177.7 (3)
C2—C1—N1—O2	2.7 (4)	N12—C19—C24—C25	1.3 (4)
C4—C3—N2—O3	−176.7 (3)	C23—C24—C25—O11	−1.7 (5)
C2—C3—N2—O3	2.9 (4)	C19—C24—C25—O11	178.5 (3)
C4—C3—N2—O4	2.8 (4)	C23—C24—C25—O12	178.5 (3)
C2—C3—N2—O4	−177.6 (3)	C19—C24—C25—O12	−1.2 (5)
C6—C5—N3—O5	−172.8 (3)	C16—C11—N11—N12	5.5 (4)
C4—C5—N3—O5	8.8 (4)	C12—C11—N11—N12	−174.9 (2)
C6—C5—N3—O6	7.8 (4)	C11—N11—N12—C19	−179.7 (2)
C4—C5—N3—O6	−170.5 (3)	C20—C19—N12—N11	−0.7 (4)
N11—C11—C12—C13	178.1 (3)	C24—C19—N12—N11	−179.7 (2)
C16—C11—C12—C13	−2.3 (4)	C13—C14—N13—C18	177.4 (3)
C11—C12—C13—C14	0.4 (4)	C15—C14—N13—C18	−2.4 (5)
C12—C13—C14—N13	−178.2 (3)	C13—C14—N13—C17	−0.4 (4)
C12—C13—C14—C15	1.6 (4)	C15—C14—N13—C17	179.8 (3)

### 1,3,5-Trinitrobenzene–2-{(E)-[4-(dimethylamino)phenyl]diazenyl}benzoic acid (1/1) (III) Hydrogen-bond geometry (Å, º)

**Table:**

*D*—H···*A*	*D*—H	H···*A*	*D*···*A*	*D*—H···*A*
C4—H4···O4^i^	0.95	2.35	3.285 (4)	169
C15—H15···O11^ii^	0.95	2.34	3.254 (4)	161
C18—H18*A*···O11^ii^	0.98	2.55	3.519 (4)	170
C20—H20···O3^iii^	0.95	2.64	3.574 (4)	167
O12—H12···N12	0.95 (6)	1.70 (6)	2.577 (3)	153 (5)
C21—H21···O2^iii^	0.95	2.56	3.469 (4)	161

Symmetry codes: (i) −*x*+1, −*y*+1, −*z*+1; (ii) −*x*, −*y*+1, −*z*+1; (iii) −*x*+1, −*y*, −*z*+1.

### 1,3,5-Trinitrobenzene–1-naphthoic acid–2-amino-5-nitropyridine (1/1/1) (IV) Crystal data

**Table:**

C_6_H_3_N_3_O_6_·C_11_H_8_O_2_·C_5_H_5_N_3_O_2_	*Z* = 2
*M**_r_* = 524.41	*F*(000) = 540
Triclinic, *P*1	*D*_x_ = 1.571 Mg m^−^^3^
Hall symbol: -P 1	Mo *K*α radiation, λ = 0.71073 Å
*a* = 7.5365 (15) Å	Cell parameters from 8623 reflections
*b* = 7.9003 (16) Å	θ = 3.1–28.3°
*c* = 19.153 (4) Å	µ = 0.13 mm^−^^1^
α = 97.580 (7)°	*T* = 173 K
β = 94.667 (6)°	Plate, brown
γ = 99.547 (7)°	0.63 × 0.33 × 0.06 mm
*V* = 1108.5 (4) Å^3^	

### 1,3,5-Trinitrobenzene–1-naphthoic acid–2-amino-5-nitropyridine (1/1/1) (IV) Data collection

**Table:**

Bruker D8 Venture Photon CCD area detector diffractometer	3368 reflections with *I* > 2σ(*I*)
Graphite monochromator	*R*_int_ = 0.034
ω scans	θ_max_ = 25.5°, θ_min_ = 3.2°
Absorption correction: multi-scan (SADABS; Sheldrick, 1996)	*h* = −9→9
*T*_min_ = 0.9, *T*_max_ = 0.95	*k* = −9→8
12982 measured reflections	*l* = −23→23
3988 independent reflections	

### 1,3,5-Trinitrobenzene–1-naphthoic acid–2-amino-5-nitropyridine (1/1/1) (IV) Refinement

**Table:**

Refinement on *F*^2^	0 constraints
Least-squares matrix: full	Hydrogen site location: mixed
*R*[*F*^2^ > 2σ(*F*^2^)] = 0.072	H atoms treated by a mixture of independent and constrained refinement
*wR*(*F*^2^) = 0.168	*w* = 1/[σ^2^(*F*_o_^2^) + 2.9593*P*] where *P* = (*F*_o_^2^ + 2*F*_c_^2^)/3
*S* = 1.22	(Δ/σ)_max_ < 0.001
3988 reflections	Δρ_max_ = 0.32 e Å^−^^3^
355 parameters	Δρ_min_ = −0.36 e Å^−^^3^
0 restraints	

### 1,3,5-Trinitrobenzene–1-naphthoic acid–2-amino-5-nitropyridine (1/1/1) (IV) Special details

**Table:**

Experimental. (SADABS; Sheldrick, 1996)
Geometry. All standard uncertainties (s.u.) (except the s.u. in the dihedral angle between two l.s. planes) are estimated using the full covariance matrix. The cell s.u. are taken into account individually in the estimation of s.u. in distances, angles and torsion angles; correlations between s.u. in cell parameters are only used when they are defined by crystal symmetry. An approximate (isotropic) treatment of cell s.u. is used for estimating s.u. involving l.s. planes.

### 1,3,5-Trinitrobenzene–1-naphthoic acid–2-amino-5-nitropyridine (1/1/1) (IV) Fractional atomic coordinates and isotropic or equivalent isotropic displacement parameters (Å^2^)

**Table:**

	*x*	*y*	*z*	*U*_iso_*/*U*_eq_	
C1	0.3489 (5)	0.8531 (5)	0.28180 (19)	0.0286 (8)	
C2	0.5027 (5)	0.7901 (5)	0.2644 (2)	0.0305 (8)	
H2	0.592398	0.774878	0.299662	0.037*	
C3	0.5190 (5)	0.7506 (5)	0.1933 (2)	0.0321 (9)	
C4	0.3924 (5)	0.7760 (5)	0.1413 (2)	0.0337 (9)	
H4	0.406294	0.747525	0.092526	0.04*	
C5	0.2448 (5)	0.8441 (5)	0.1624 (2)	0.0309 (8)	
C6	0.2177 (5)	0.8818 (5)	0.23294 (19)	0.0287 (8)	
H6	0.113733	0.925419	0.246843	0.034*	
N1	0.3220 (5)	0.8876 (4)	0.35735 (17)	0.0340 (8)	
N2	0.6777 (5)	0.6813 (5)	0.1725 (2)	0.0445 (9)	
N3	0.1080 (5)	0.8742 (5)	0.10842 (18)	0.0416 (9)	
O1	0.1697 (4)	0.8928 (4)	0.37245 (15)	0.0441 (7)	
O2	0.4547 (4)	0.9063 (5)	0.40103 (15)	0.0521 (9)	
O3	0.7923 (4)	0.6648 (5)	0.2201 (2)	0.0562 (9)	
O4	0.6891 (5)	0.6423 (5)	0.10927 (19)	0.0617 (10)	
O5	0.1245 (5)	0.8221 (6)	0.04677 (17)	0.0680 (11)	
O6	−0.0122 (4)	0.9495 (5)	0.12813 (17)	0.0520 (8)	
C11	0.2801 (5)	0.3592 (5)	0.23195 (19)	0.0283 (8)	
C12	0.1324 (5)	0.4164 (5)	0.2022 (2)	0.0339 (9)	
H12	0.0557	0.466551	0.232661	0.041*	
C13	0.0906 (6)	0.4036 (6)	0.1289 (2)	0.0413 (10)	
H13	−0.013342	0.442495	0.109979	0.05*	
C14	0.2039 (6)	0.3333 (6)	0.0846 (2)	0.0423 (11)	
H14	0.176484	0.322585	0.034787	0.051*	
C15	0.3602 (5)	0.2768 (5)	0.11240 (19)	0.0333 (9)	
C16	0.4790 (6)	0.2077 (6)	0.0674 (2)	0.0427 (11)	
H16	0.451417	0.196379	0.017572	0.051*	
C17	0.6309 (6)	0.1573 (6)	0.0937 (2)	0.0465 (11)	
H17	0.709603	0.112982	0.06254	0.056*	
C18	0.6715 (5)	0.1709 (5)	0.1671 (2)	0.0370 (10)	
H18	0.778609	0.136036	0.185356	0.044*	
C19	0.5604 (5)	0.2332 (5)	0.2127 (2)	0.0309 (8)	
H19	0.590396	0.240016	0.262266	0.037*	
C20	0.3986 (5)	0.2887 (5)	0.18715 (19)	0.0296 (8)	
C21	0.3071 (5)	0.3680 (5)	0.31063 (19)	0.0293 (8)	
O7	0.2603 (4)	0.5052 (4)	0.34527 (15)	0.0370 (7)	
O8	0.3642 (4)	0.2557 (4)	0.33988 (14)	0.0385 (7)	
H5A	0.309 (7)	0.226 (7)	0.440 (3)	0.058 (15)*	
H5B	0.256 (5)	0.144 (6)	0.506 (2)	0.030 (11)*	
H7	0.270 (7)	0.503 (7)	0.395 (3)	0.063 (15)*	
C22	0.2465 (5)	0.3802 (5)	0.51944 (18)	0.0265 (8)	
C23	0.1956 (5)	0.3906 (5)	0.58930 (19)	0.0296 (8)	
H23	0.183191	0.291092	0.612665	0.036*	
C24	0.1651 (5)	0.5429 (5)	0.62225 (19)	0.0309 (9)	
H24	0.132639	0.553261	0.669335	0.037*	
C25	0.1822 (5)	0.6859 (5)	0.58541 (19)	0.0272 (8)	
C26	0.2293 (5)	0.6680 (5)	0.51770 (19)	0.0295 (8)	
H26	0.240027	0.766188	0.493543	0.035*	
N4	0.2607 (4)	0.5197 (4)	0.48431 (15)	0.0290 (7)	
N5	0.2777 (5)	0.2340 (4)	0.48463 (19)	0.0353 (8)	
N6	0.1470 (4)	0.8518 (4)	0.61748 (17)	0.0325 (7)	
O9	0.1578 (4)	0.9701 (4)	0.58321 (17)	0.0439 (7)	
O10	0.1098 (5)	0.8655 (4)	0.67946 (15)	0.0508 (8)	

### 1,3,5-Trinitrobenzene–1-naphthoic acid–2-amino-5-nitropyridine (1/1/1) (IV) Atomic displacement parameters (Å^2^)

**Table:**

	*U*^11^	*U*^22^	*U*^33^	*U*^12^	*U*^13^	*U*^23^
C1	0.0303 (19)	0.025 (2)	0.0300 (19)	0.0012 (15)	0.0051 (15)	0.0064 (15)
C2	0.0284 (19)	0.026 (2)	0.038 (2)	0.0046 (16)	0.0043 (16)	0.0101 (16)
C3	0.0267 (19)	0.025 (2)	0.045 (2)	0.0021 (16)	0.0119 (17)	0.0048 (17)
C4	0.034 (2)	0.031 (2)	0.034 (2)	−0.0036 (17)	0.0090 (16)	0.0040 (17)
C5	0.0273 (19)	0.030 (2)	0.033 (2)	−0.0025 (16)	0.0025 (15)	0.0057 (16)
C6	0.0250 (18)	0.0252 (19)	0.036 (2)	0.0028 (15)	0.0065 (15)	0.0047 (16)
N1	0.042 (2)	0.0284 (18)	0.0328 (18)	0.0076 (15)	0.0059 (15)	0.0074 (14)
N2	0.035 (2)	0.037 (2)	0.064 (3)	0.0037 (16)	0.0163 (18)	0.0109 (19)
N3	0.037 (2)	0.050 (2)	0.034 (2)	−0.0059 (17)	0.0021 (15)	0.0095 (17)
O1	0.0461 (18)	0.052 (2)	0.0390 (16)	0.0127 (15)	0.0175 (14)	0.0090 (14)
O2	0.0512 (19)	0.069 (2)	0.0340 (16)	0.0103 (17)	−0.0051 (14)	0.0065 (15)
O3	0.0378 (18)	0.056 (2)	0.083 (3)	0.0206 (15)	0.0153 (17)	0.0201 (19)
O4	0.052 (2)	0.068 (3)	0.064 (2)	0.0079 (18)	0.0286 (17)	−0.0023 (19)
O5	0.058 (2)	0.107 (3)	0.0313 (18)	0.004 (2)	−0.0025 (15)	0.0035 (18)
O6	0.0405 (18)	0.066 (2)	0.0508 (19)	0.0108 (16)	0.0000 (15)	0.0138 (17)
C11	0.0286 (19)	0.024 (2)	0.0307 (19)	0.0004 (15)	0.0074 (15)	0.0017 (15)
C12	0.030 (2)	0.033 (2)	0.039 (2)	0.0021 (17)	0.0073 (17)	0.0092 (17)
C13	0.033 (2)	0.047 (3)	0.040 (2)	−0.0048 (19)	−0.0023 (18)	0.0105 (19)
C14	0.044 (2)	0.046 (3)	0.030 (2)	−0.006 (2)	−0.0002 (18)	0.0029 (19)
C15	0.035 (2)	0.034 (2)	0.0260 (19)	−0.0062 (17)	0.0064 (16)	−0.0008 (16)
C16	0.055 (3)	0.041 (3)	0.030 (2)	0.002 (2)	0.0142 (19)	−0.0001 (18)
C17	0.049 (3)	0.044 (3)	0.044 (2)	0.000 (2)	0.018 (2)	−0.001 (2)
C18	0.032 (2)	0.035 (2)	0.041 (2)	−0.0008 (17)	0.0113 (17)	−0.0005 (18)
C19	0.0294 (19)	0.026 (2)	0.035 (2)	0.0004 (16)	0.0047 (16)	0.0007 (16)
C20	0.032 (2)	0.025 (2)	0.0282 (19)	−0.0052 (16)	0.0064 (15)	0.0018 (15)
C21	0.0298 (19)	0.027 (2)	0.031 (2)	0.0026 (16)	0.0085 (15)	0.0050 (17)
O7	0.0569 (18)	0.0299 (16)	0.0279 (15)	0.0144 (13)	0.0103 (13)	0.0055 (12)
O8	0.0495 (17)	0.0396 (17)	0.0307 (14)	0.0163 (14)	0.0107 (12)	0.0064 (13)
C22	0.0257 (18)	0.026 (2)	0.0279 (18)	0.0047 (15)	0.0000 (14)	0.0045 (15)
C23	0.0316 (19)	0.032 (2)	0.0265 (19)	0.0071 (16)	0.0016 (15)	0.0087 (16)
C24	0.033 (2)	0.034 (2)	0.0249 (18)	0.0044 (17)	0.0033 (15)	0.0053 (16)
C25	0.0253 (18)	0.028 (2)	0.0278 (18)	0.0037 (15)	0.0020 (14)	0.0022 (15)
C26	0.033 (2)	0.023 (2)	0.032 (2)	0.0046 (16)	0.0036 (16)	0.0052 (16)
N4	0.0364 (17)	0.0246 (17)	0.0265 (16)	0.0041 (13)	0.0071 (13)	0.0043 (13)
N5	0.057 (2)	0.0199 (18)	0.0309 (18)	0.0082 (16)	0.0117 (16)	0.0045 (15)
N6	0.0298 (17)	0.0297 (19)	0.0373 (19)	0.0056 (14)	0.0041 (14)	0.0019 (15)
O9	0.0505 (18)	0.0277 (16)	0.0605 (19)	0.0122 (13)	0.0210 (15)	0.0159 (14)
O10	0.075 (2)	0.051 (2)	0.0318 (16)	0.0285 (17)	0.0119 (15)	0.0012 (14)

### 1,3,5-Trinitrobenzene–1-naphthoic acid–2-amino-5-nitropyridine (1/1/1) (IV) Geometric parameters (Å, º)

**Table:**

C1—C6	1.373 (5)	C15—C20	1.425 (5)
C1—C2	1.382 (5)	C16—C17	1.354 (7)
C1—N1	1.474 (5)	C16—H16	0.95
C2—C3	1.376 (5)	C17—C18	1.400 (6)
C2—H2	0.95	C17—H17	0.95
C3—C4	1.380 (6)	C18—C19	1.360 (5)
C3—N2	1.458 (5)	C18—H18	0.95
C4—C5	1.380 (6)	C19—C20	1.434 (6)
C4—H4	0.95	C19—H19	0.95
C5—C6	1.383 (5)	C21—O8	1.227 (5)
C5—N3	1.470 (5)	C21—O7	1.309 (5)
C6—H6	0.95	O7—H7	0.95 (5)
N1—O1	1.212 (4)	C22—N5	1.321 (5)
N1—O2	1.227 (4)	C22—N4	1.359 (5)
N2—O4	1.222 (5)	C22—C23	1.418 (5)
N2—O3	1.240 (5)	C23—C24	1.347 (6)
N3—O5	1.221 (5)	C23—H23	0.95
N3—O6	1.222 (5)	C24—C25	1.403 (5)
C11—C12	1.378 (5)	C24—H24	0.95
C11—C20	1.417 (5)	C25—C26	1.368 (5)
C11—C21	1.495 (5)	C25—N6	1.447 (5)
C12—C13	1.400 (6)	C26—N4	1.325 (5)
C12—H12	0.95	C26—H26	0.95
C13—C14	1.380 (6)	N5—H5A	0.90 (5)
C13—H13	0.95	N5—H5B	0.86 (4)
C14—C15	1.416 (6)	N6—O9	1.207 (4)
C14—H14	0.95	N6—O10	1.237 (4)
C15—C16	1.416 (6)		
			
C6—C1—C2	124.0 (3)	C17—C16—C15	121.7 (4)
C6—C1—N1	117.9 (3)	C17—C16—H16	119.2
C2—C1—N1	118.1 (3)	C15—C16—H16	119.2
C3—C2—C1	116.6 (4)	C16—C17—C18	119.7 (4)
C3—C2—H2	121.7	C16—C17—H17	120.2
C1—C2—H2	121.7	C18—C17—H17	120.2
C2—C3—C4	122.5 (4)	C19—C18—C17	121.1 (4)
C2—C3—N2	118.5 (4)	C19—C18—H18	119.4
C4—C3—N2	119.0 (4)	C17—C18—H18	119.4
C5—C4—C3	117.8 (4)	C18—C19—C20	121.0 (4)
C5—C4—H4	121.1	C18—C19—H19	119.5
C3—C4—H4	121.1	C20—C19—H19	119.5
C4—C5—C6	122.5 (4)	C11—C20—C15	118.9 (4)
C4—C5—N3	119.3 (3)	C11—C20—C19	123.7 (3)
C6—C5—N3	118.2 (4)	C15—C20—C19	117.3 (4)
C1—C6—C5	116.5 (4)	O8—C21—O7	123.2 (3)
C1—C6—H6	121.7	O8—C21—C11	123.2 (4)
C5—C6—H6	121.7	O7—C21—C11	113.5 (3)
O1—N1—O2	124.1 (3)	C21—O7—H7	112 (3)
O1—N1—C1	118.0 (3)	N5—C22—N4	116.8 (3)
O2—N1—C1	117.9 (3)	N5—C22—C23	121.9 (4)
O4—N2—O3	124.1 (4)	N4—C22—C23	121.3 (3)
O4—N2—C3	118.1 (4)	C24—C23—C22	119.4 (4)
O3—N2—C3	117.8 (4)	C24—C23—H23	120.3
O5—N3—O6	124.9 (4)	C22—C23—H23	120.3
O5—N3—C5	116.9 (4)	C23—C24—C25	118.4 (3)
O6—N3—C5	118.1 (3)	C23—C24—H24	120.8
C12—C11—C20	119.2 (3)	C25—C24—H24	120.8
C12—C11—C21	118.9 (3)	C26—C25—C24	119.7 (4)
C20—C11—C21	121.9 (3)	C26—C25—N6	119.4 (3)
C11—C12—C13	122.8 (4)	C24—C25—N6	120.9 (3)
C11—C12—H12	118.6	N4—C26—C25	122.9 (4)
C13—C12—H12	118.6	N4—C26—H26	118.5
C14—C13—C12	118.5 (4)	C25—C26—H26	118.5
C14—C13—H13	120.7	C26—N4—C22	118.2 (3)
C12—C13—H13	120.7	C22—N5—H5A	123 (3)
C13—C14—C15	121.1 (4)	C22—N5—H5B	116 (3)
C13—C14—H14	119.5	H5A—N5—H5B	121 (4)
C15—C14—H14	119.5	O9—N6—O10	123.2 (3)
C14—C15—C16	121.4 (4)	O9—N6—C25	118.9 (3)
C14—C15—C20	119.4 (4)	O10—N6—C25	117.9 (3)
C16—C15—C20	119.1 (4)		
			
C6—C1—C2—C3	−1.8 (6)	C15—C16—C17—C18	1.0 (7)
N1—C1—C2—C3	176.7 (3)	C16—C17—C18—C19	0.3 (7)
C1—C2—C3—C4	1.7 (6)	C17—C18—C19—C20	−0.6 (6)
C1—C2—C3—N2	−179.1 (3)	C12—C11—C20—C15	−0.8 (5)
C2—C3—C4—C5	0.2 (6)	C21—C11—C20—C15	177.1 (3)
N2—C3—C4—C5	−179.1 (3)	C12—C11—C20—C19	177.0 (4)
C3—C4—C5—C6	−2.1 (6)	C21—C11—C20—C19	−5.1 (5)
C3—C4—C5—N3	179.5 (3)	C14—C15—C20—C11	−0.8 (5)
C2—C1—C6—C5	0.1 (6)	C16—C15—C20—C11	179.5 (3)
N1—C1—C6—C5	−178.4 (3)	C14—C15—C20—C19	−178.8 (4)
C4—C5—C6—C1	1.9 (5)	C16—C15—C20—C19	1.6 (5)
N3—C5—C6—C1	−179.6 (3)	C18—C19—C20—C11	−178.2 (4)
C6—C1—N1—O1	19.8 (5)	C18—C19—C20—C15	−0.3 (5)
C2—C1—N1—O1	−158.8 (4)	C12—C11—C21—O8	142.1 (4)
C6—C1—N1—O2	−161.4 (4)	C20—C11—C21—O8	−35.8 (5)
C2—C1—N1—O2	20.0 (5)	C12—C11—C21—O7	−36.5 (5)
C2—C3—N2—O4	178.1 (4)	C20—C11—C21—O7	145.6 (3)
C4—C3—N2—O4	−2.6 (5)	N5—C22—C23—C24	−179.8 (4)
C2—C3—N2—O3	−1.5 (5)	N4—C22—C23—C24	−1.7 (5)
C4—C3—N2—O3	177.8 (4)	C22—C23—C24—C25	1.0 (5)
C4—C5—N3—O5	6.4 (5)	C23—C24—C25—C26	−0.1 (5)
C6—C5—N3—O5	−172.1 (4)	C23—C24—C25—N6	178.6 (3)
C4—C5—N3—O6	−173.2 (4)	C24—C25—C26—N4	−0.1 (6)
C6—C5—N3—O6	8.3 (5)	N6—C25—C26—N4	−178.8 (3)
C20—C11—C12—C13	1.8 (6)	C25—C26—N4—C22	−0.5 (5)
C21—C11—C12—C13	−176.1 (4)	N5—C22—N4—C26	179.7 (3)
C11—C12—C13—C14	−1.0 (6)	C23—C22—N4—C26	1.5 (5)
C12—C13—C14—C15	−0.7 (6)	C26—C25—N6—O9	1.0 (5)
C13—C14—C15—C16	−178.7 (4)	C24—C25—N6—O9	−177.7 (3)
C13—C14—C15—C20	1.6 (6)	C26—C25—N6—O10	−178.0 (3)
C14—C15—C16—C17	178.4 (4)	C24—C25—N6—O10	3.4 (5)
C20—C15—C16—C17	−1.9 (6)		

### 1,3,5-Trinitrobenzene–1-naphthoic acid–2-amino-5-nitropyridine (1/1/1) (IV) Hydrogen-bond geometry (Å, º)

**Table:**

*D*—H···*A*	*D*—H	H···*A*	*D*···*A*	*D*—H···*A*
N5—H5*A*···O8	0.90 (5)	2.03 (5)	2.919 (5)	168 (5)
N5—H5*B*···O9^i^	0.86 (4)	2.24 (4)	3.069 (5)	161 (4)
O7—H7···N4	0.95 (5)	1.71 (5)	2.650 (4)	171 (5)
C23—H23···O9^i^	0.95	2.5	3.272 (5)	139
C26—H26···O1	0.95	2.69	3.530 (5)	147
C26—H26···O2	0.95	2.72	3.477 (5)	138

Symmetry code: (i) *x*, *y*−1, *z*.
