# Supplementary material for: Binary and ternary charge-transfer complexes using 1,3,5-tri­nitro­benzene
Source: Acta Crystallogr E Crystallogr Commun. 2018 Jan 9;74(Pt 2):113–8. doi: 10.1107/S2056989018000245 (PMC5956318; doi:10.1107/S2056989018000245)

ELECTRONIC SUPPLEMENTARY INFORMATION – FINGERPRINT PLOTS

(I)– (IV)

Structure (I)

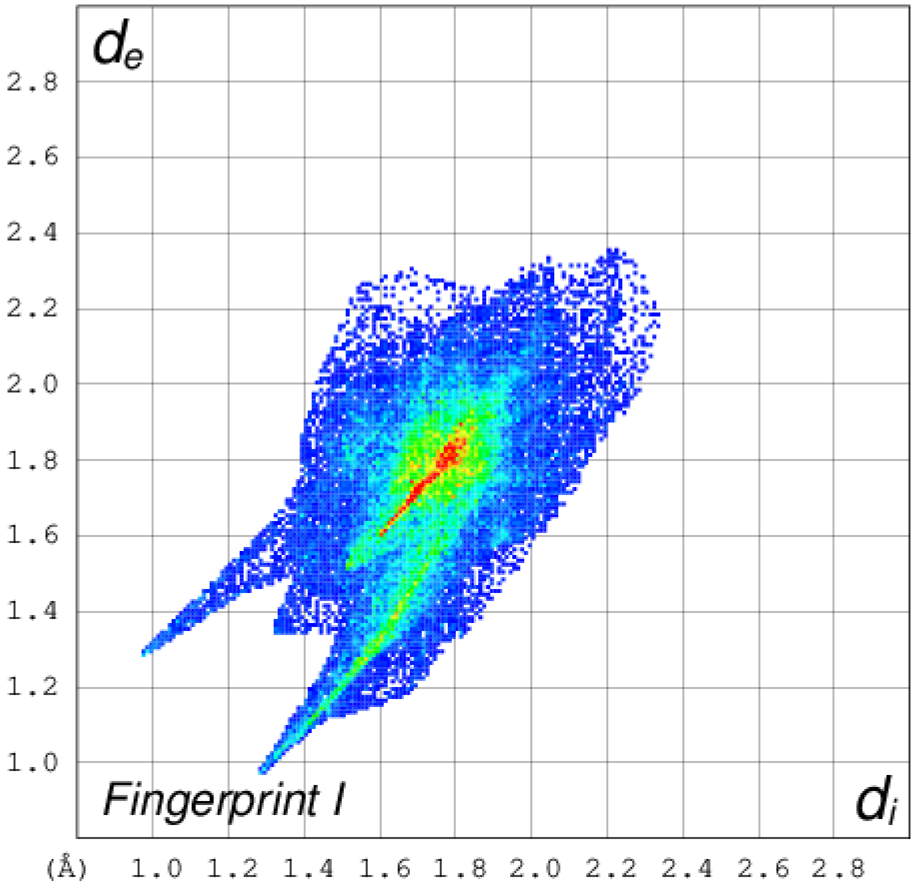

Fingerprint C•••C 12.0%

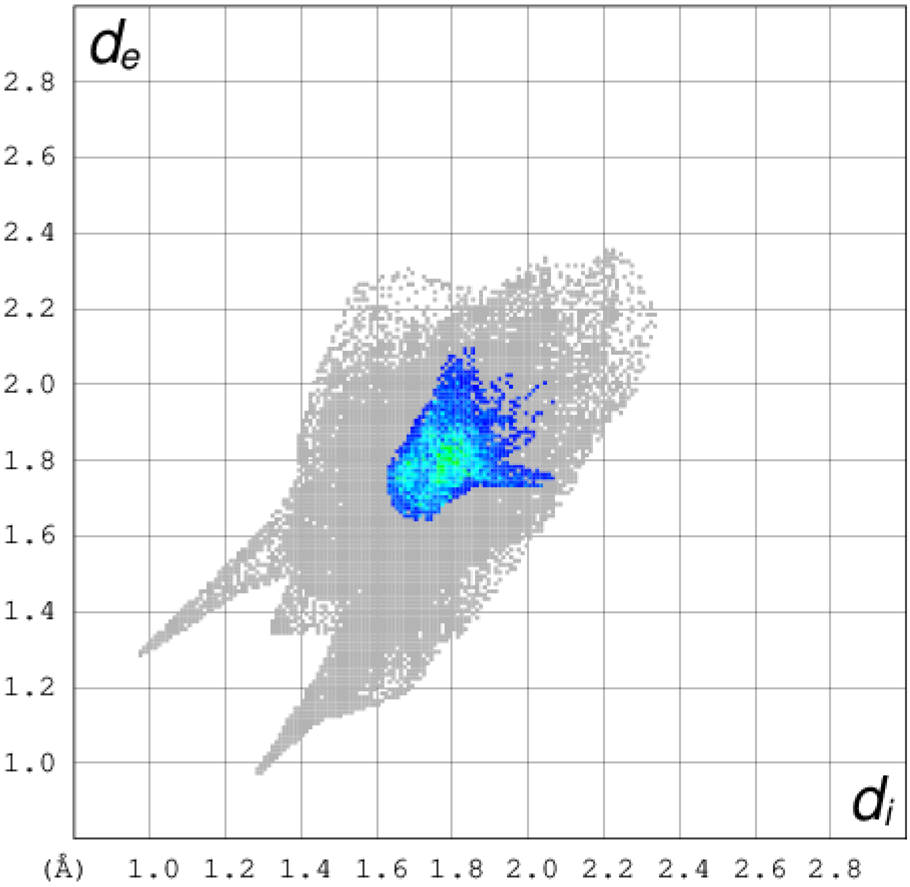

Fingerprint H•••H 10.7%

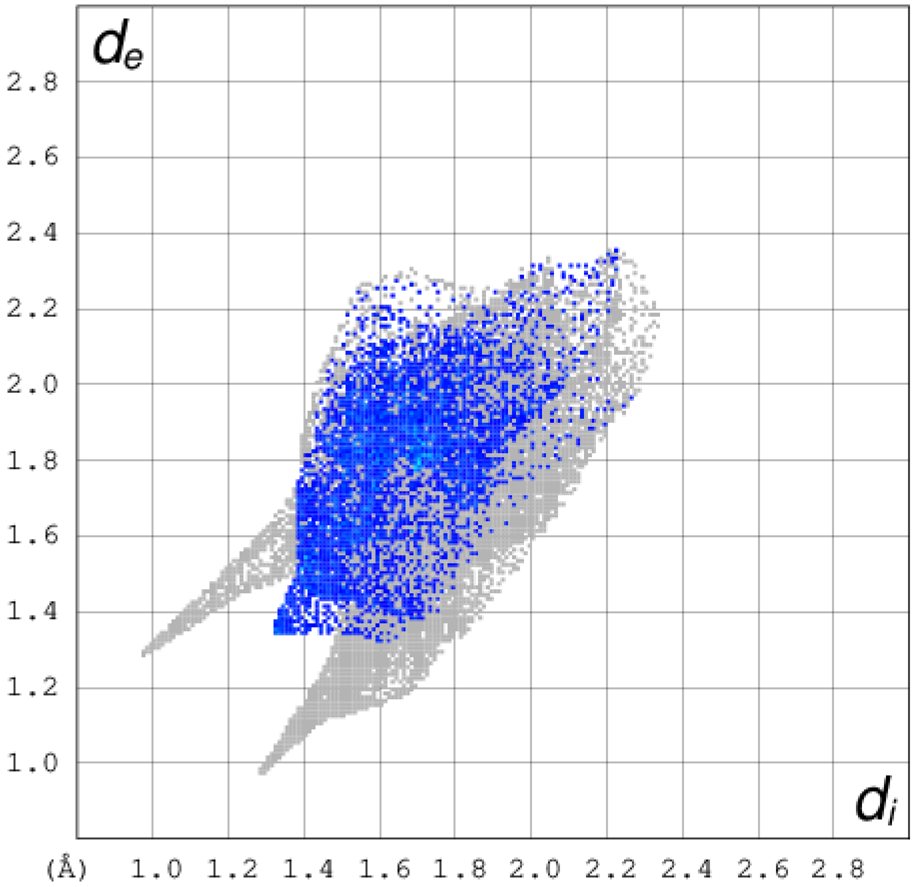

Fingerprint C•••H 1.5%

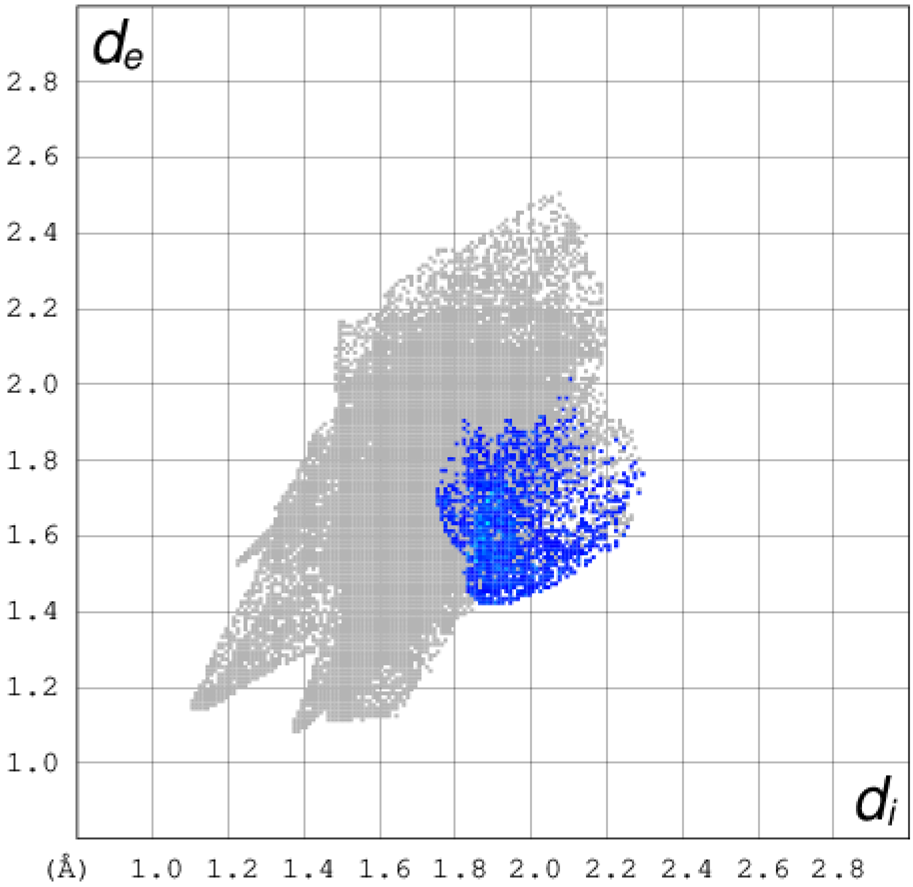

Structure (II)

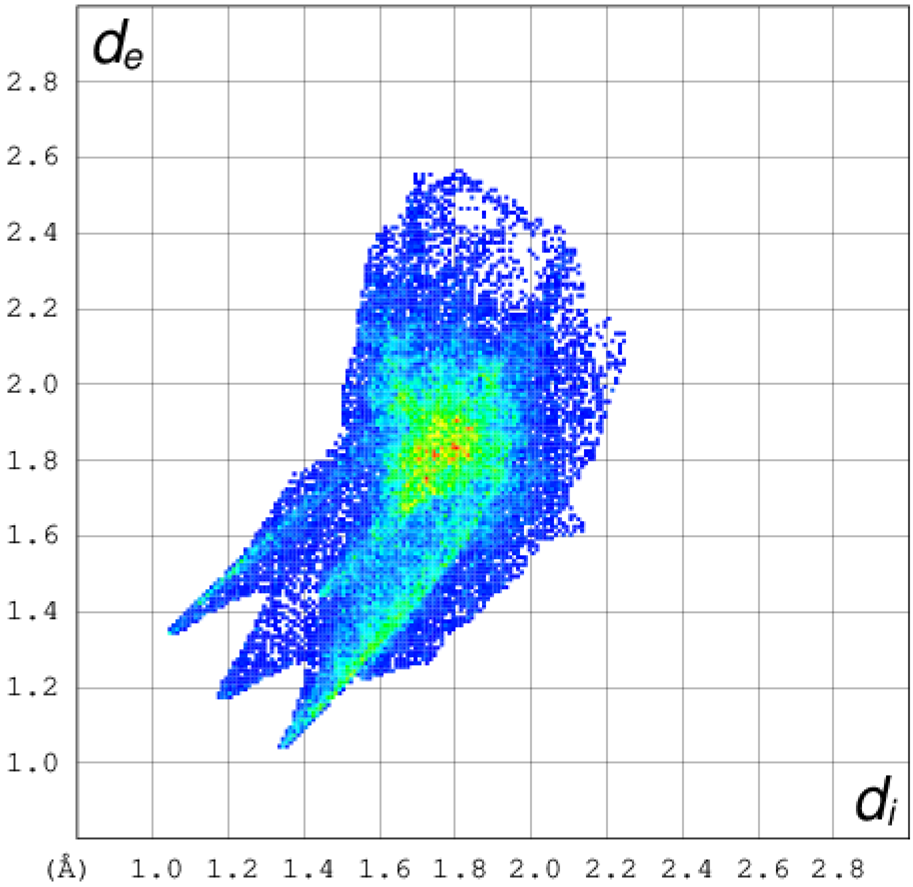

Fingerprint C•••C 12.6%

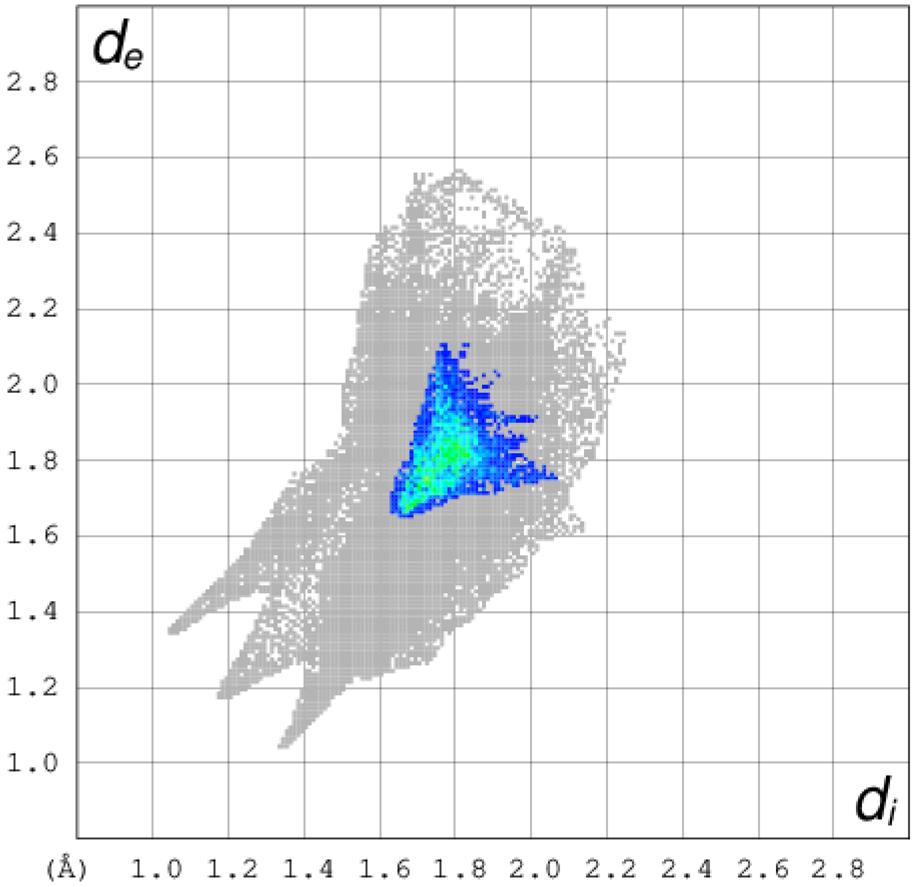

Fingerprint H•••H 6.3%

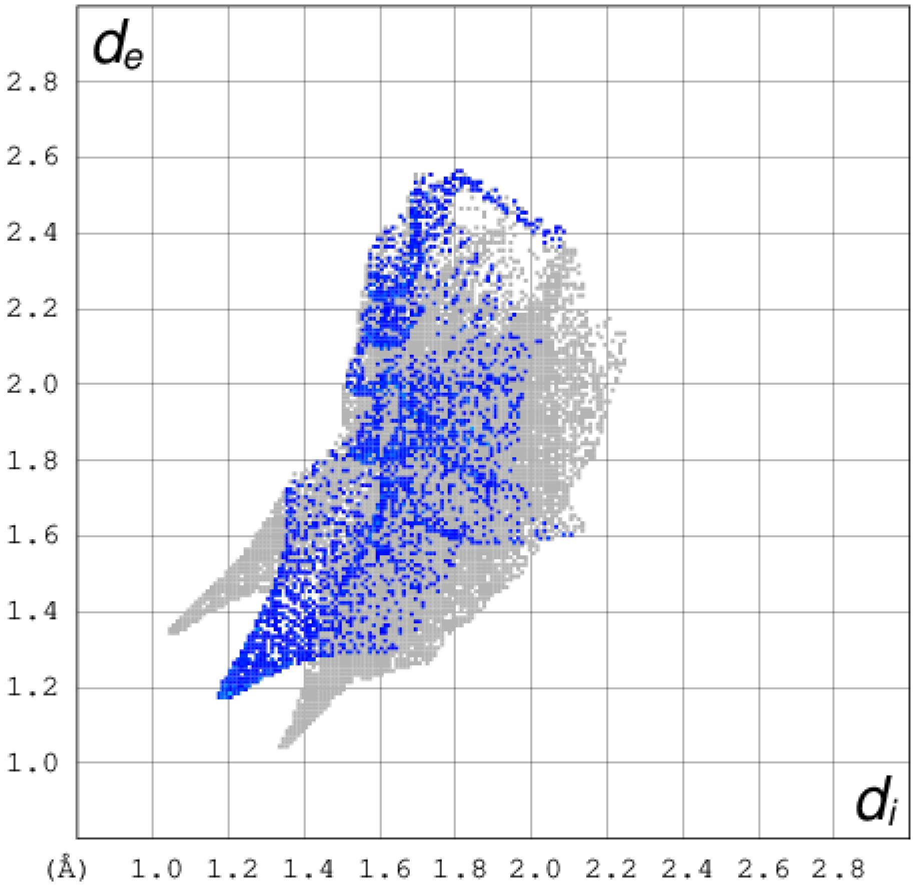

Fingerprint C•••H 0.9%

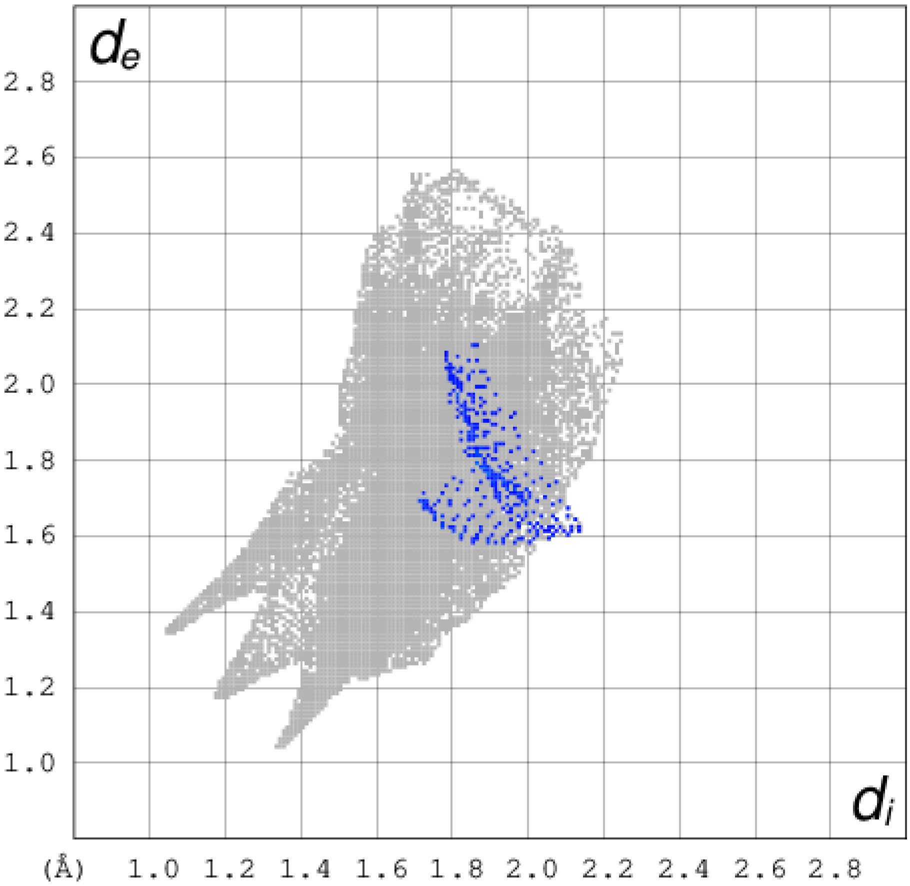

Structure (III)

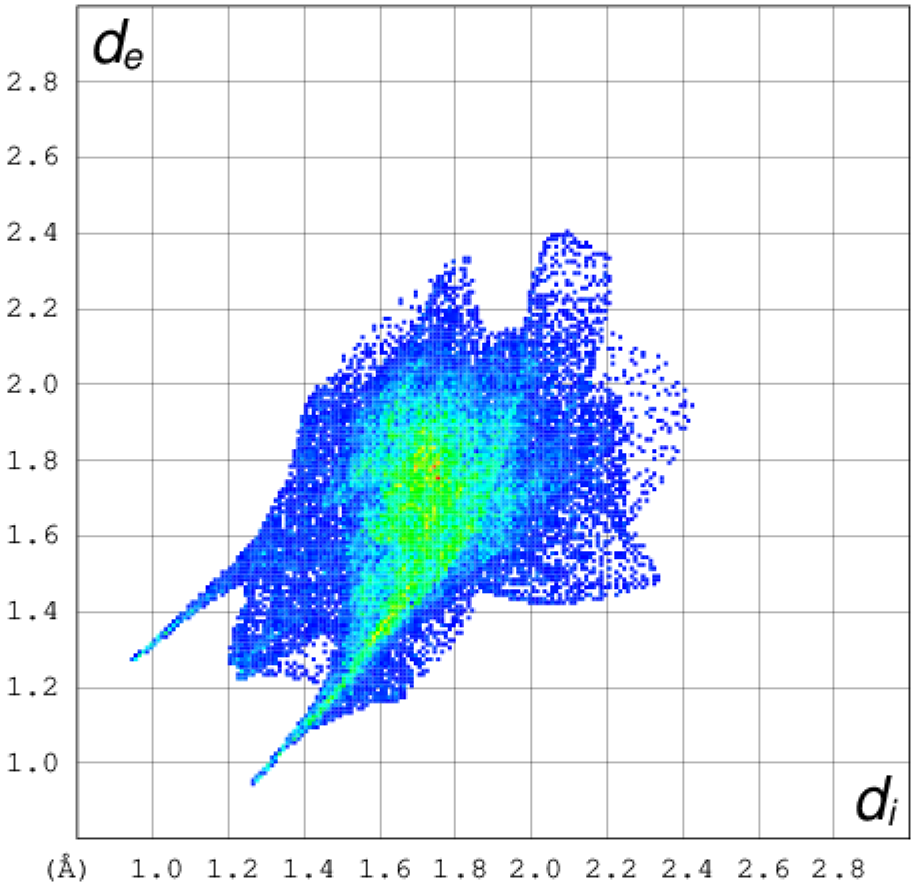

Fingerprint C•••C 4.4%

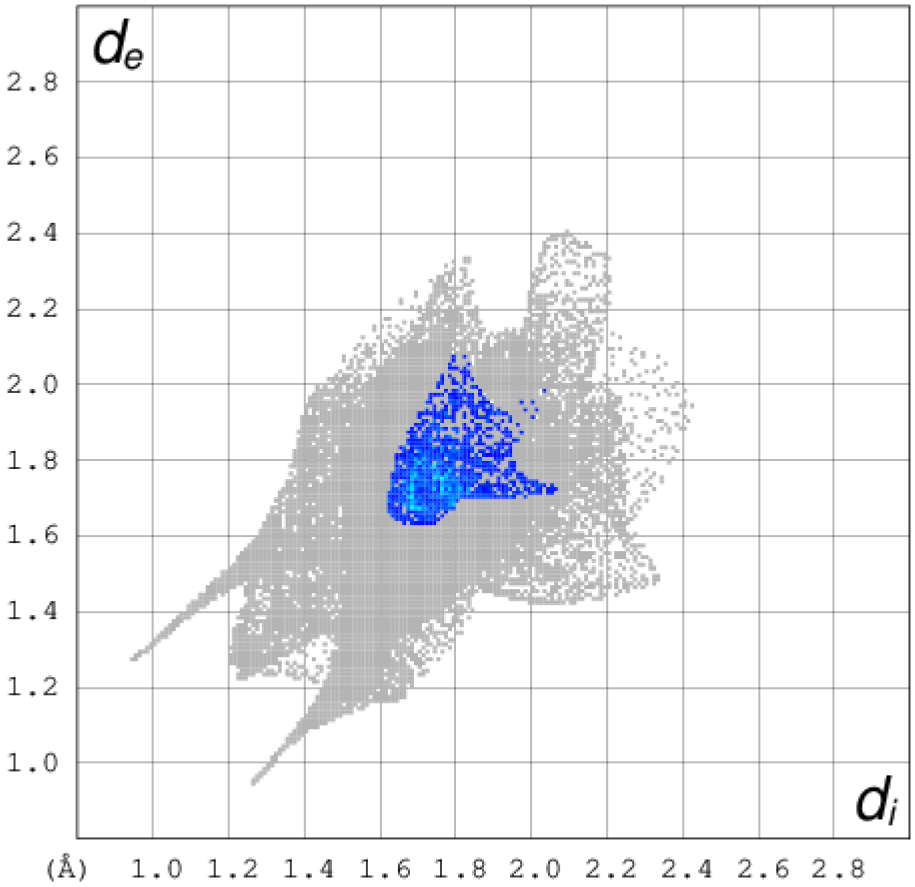

Fingerprint H•••H 11.0%

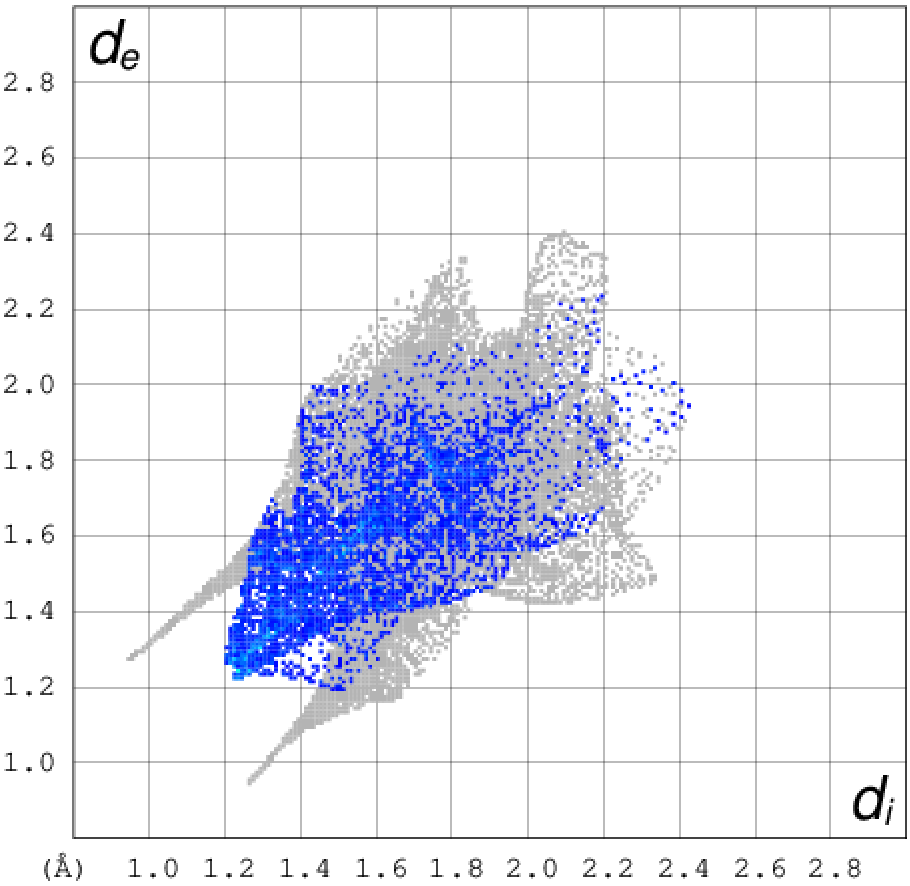

Fingerprint C•••H 5.4%

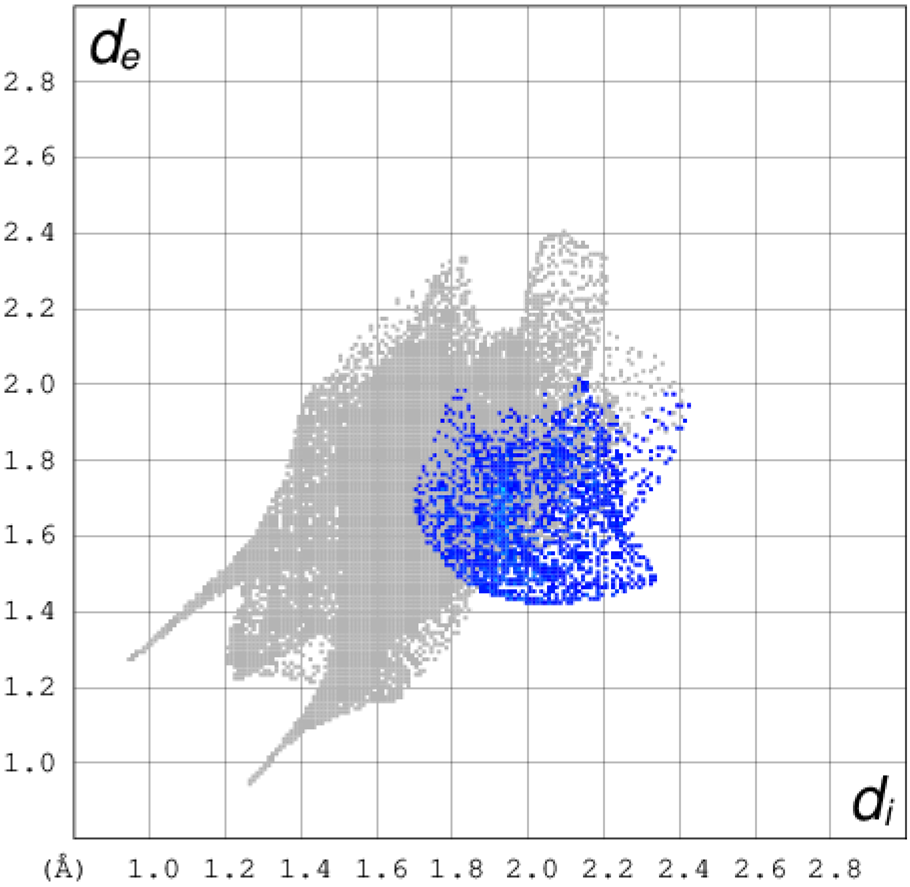

Structure (IV)

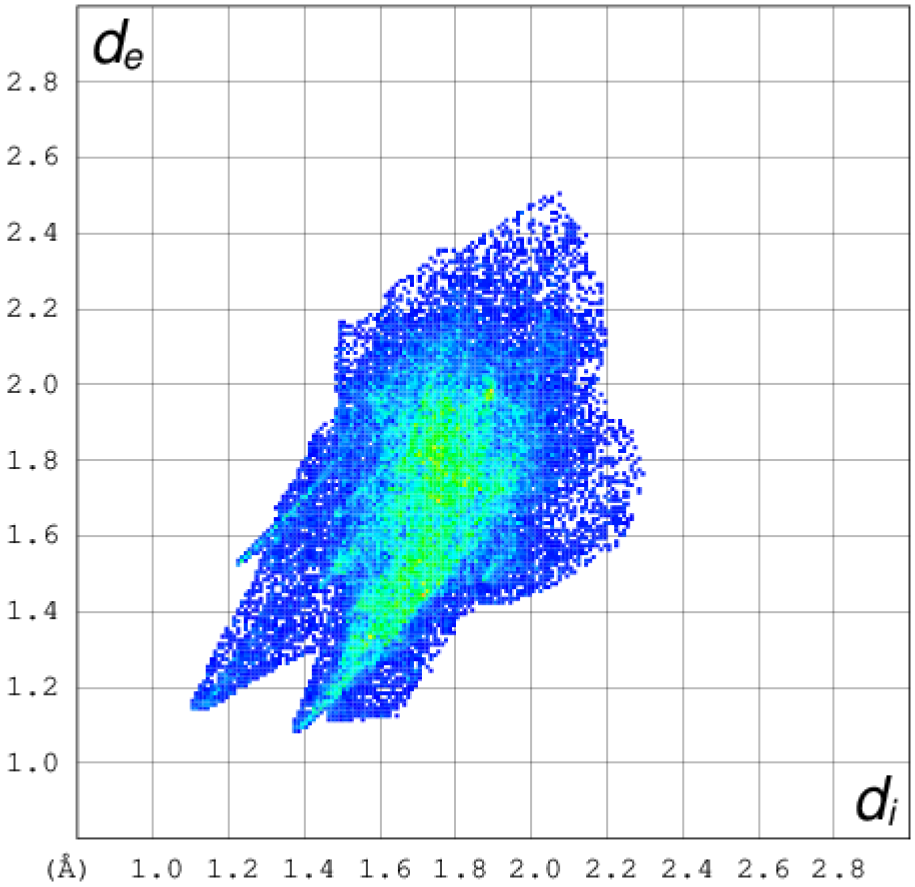

Fingerprint C•••C 7.5%

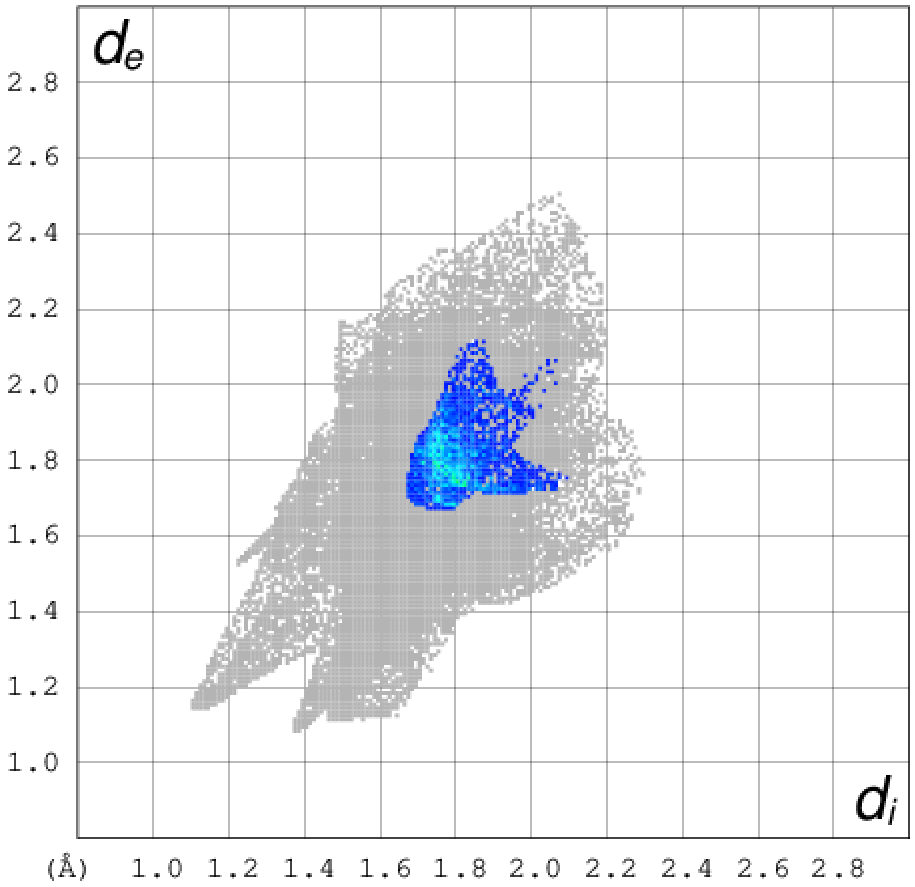

Fingerprint H•••H 8.8%

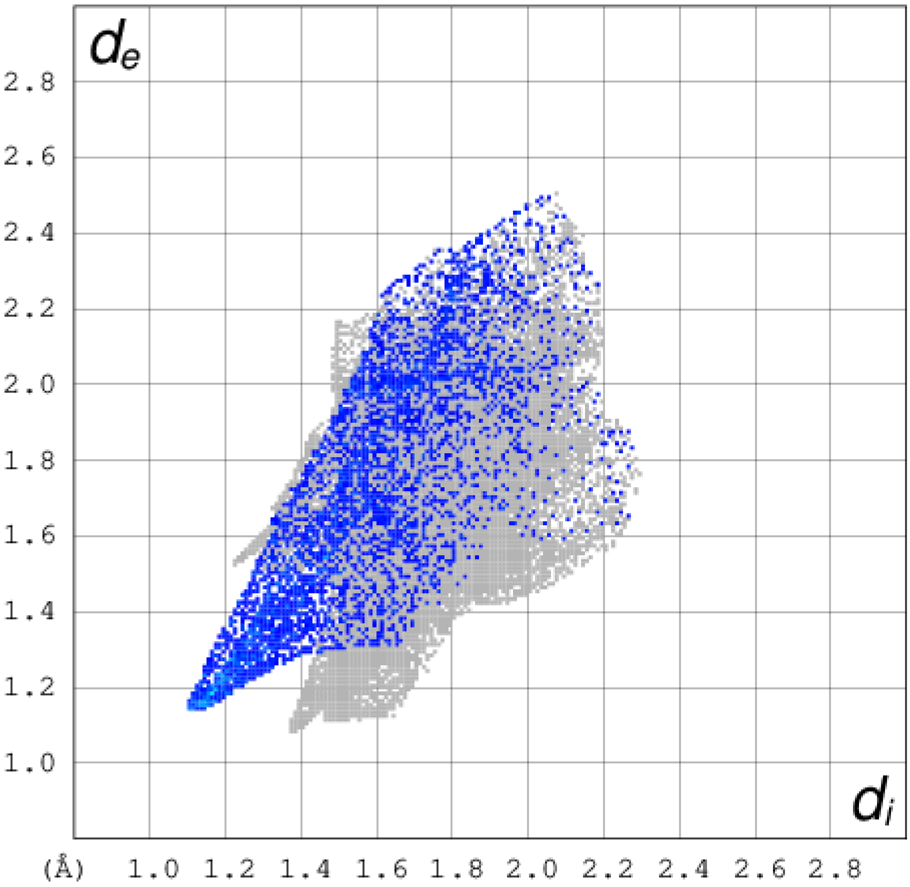

Fingerprint C•••H 4.6%

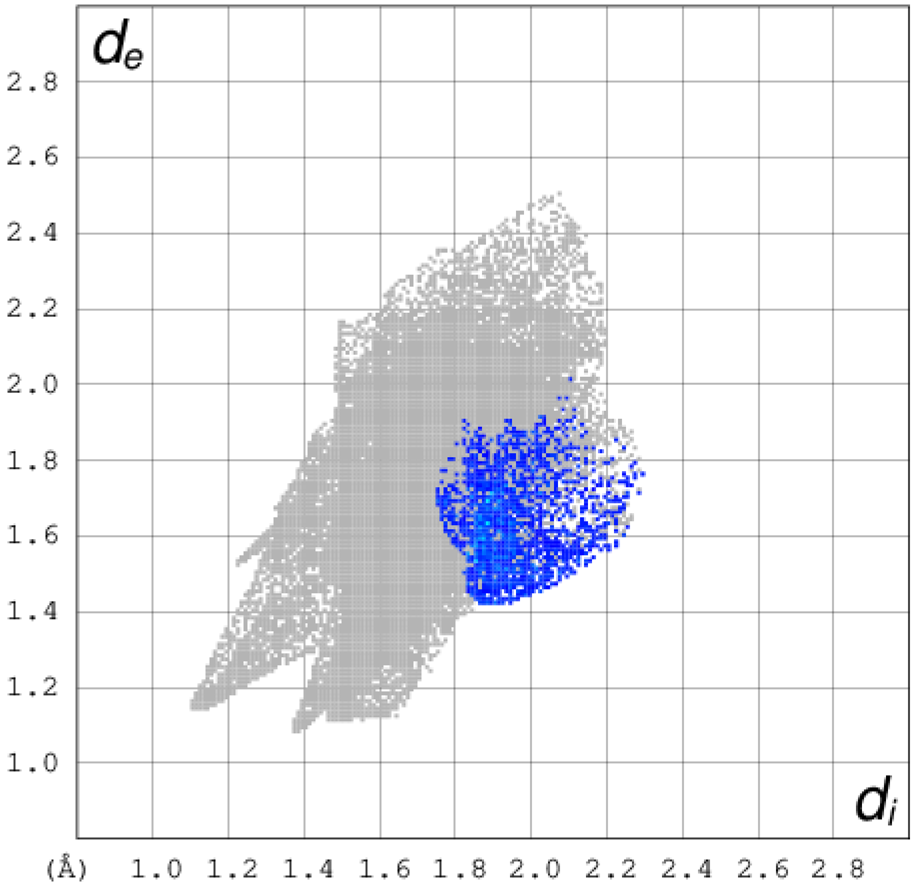

Supplement: Supplementary file 6 [file e-74-00113-sup6.pdf]
